# The Energy Content and Composition of Meals Consumed after an Overnight Fast and Their Effects on Diet Induced Thermogenesis: A Systematic Review, Meta-Analyses and Meta-Regressions

**DOI:** 10.3390/nu8110670

**Published:** 2016-10-25

**Authors:** Angelica Quatela, Robin Callister, Amanda Patterson, Lesley MacDonald-Wicks

**Affiliations:** 1 Discipline of Nutrition and Dietetics, School of Health Sciences, The University of Newcastle, University Drive, Callaghan 2308, NSW, Australia; angelica.quatela@uon.edu.au (A.Q.); amanda.patterson@newcastle.edu.au (A.P.); 2 Priority Research Centre for Physical Activity and Nutrition, School of Biomedical Sciences and Pharmacy, The University of Newcastle, University Drive, Callaghan 2308, NSW, Australia; robin.callister@newcastle.edu.au

**Keywords:** breakfast, meal, overnight fast, energy intake, macronutrient, diet-induced thermogenesis, thermic effect of food, meal-induced thermogenesis, resting metabolic rate

## Abstract

This systematic review investigated the effects of differing energy intakes, macronutrient compositions, and eating patterns of meals consumed after an overnight fast on Diet Induced Thermogenesis (DIT). The initial search identified 2482 records; 26 papers remained once duplicates were removed and inclusion criteria were applied. Studies (*n* = 27) in the analyses were randomized crossover designs comparing the effects of two or more eating events on DIT. Higher energy intake increased DIT; in a mixed model meta-regression, for every 100 kJ increase in energy intake, DIT increased by 1.1 kJ/h (*p* < 0.001). Meals with a high protein or carbohydrate content had a higher DIT than high fat, although this effect was not always significant. Meals with medium chain triglycerides had a significantly higher DIT than long chain triglycerides (meta-analysis, *p* = 0.002). Consuming the same meal as a single bolus eating event compared to multiple small meals or snacks was associated with a significantly higher DIT (meta-analysis, *p* = 0.02). Unclear or inconsistent findings were found by comparing the consumption of meals quickly or slowly, and palatability was not significantly associated with DIT. These findings indicate that the magnitude of the increase in DIT is influenced by the energy intake, macronutrient composition, and eating pattern of the meal.

## 1. Introduction

The meal consumed after an overnight fast, generally referred to as breakfast, is often described as ‘the most important meal of the day’ [1] as it is believed to contribute to good health and nutrition by providing essential nutrients early in the day [2]. Skipping breakfast is associated with increased weight gain and obesity, suggesting that breakfast may be protective against weight gain [1,3]. Among the explanations for this protective effect of breakfast are that it stimulates the body’s metabolism because it breaks the overnight fast [4], potentially contributing to increased total daily energy expenditure. The extent of this effect would depend on the diet induced thermogenesis (DIT) response to the meal consumed. Evidence supporting this proposal is limited and contradictory [5,6]. Alternatively, eating breakfast may result in decreased energy consumption during the rest of the day, however the evidence available from previous trials in this area is also limited and contradictory [1].

Obesity is a major public health concern internationally with an estimated 13% and 39% of adults worldwide being obese or overweight respectively [7], and 63% being either overweight or obese in Australia [8]. Breakfast is often advocated as a strategy to prevent weight gain. However, a recent review [3] and a separate meta-analysis [1] found that there is very limited evidence regarding the effects of breakfast on preventing weight gain [1,3], and as most of the studies conducted have only been observational, there has been little investigation into the mechanisms by which breakfast may exert effects on preventing obesity [1]. Both of these reviews found insufficient evidence to support consumption of breakfast for obesity prevention and suggested that further research in this area is required [1,3].

Given the suggested importance of breakfast in the health arena, there are surprisingly few systematic reviews (SRs) consolidating the evidence of its effects on obesity prevention [1,9,10] and no SR and/or meta-analyses have investigated the effect of consuming breakfast on accelerating DIT, which may contribute to a reduced risk of weight gain. Most studies investigating the effects of food on DIT investigate these effects after an overnight fast. Therefore these studies provide insights into the possible effects of breakfast on DIT although the meals used in these studies may not be typical of those eaten as breakfast and their goal may not have been to investigate the effects of breakfast.

No SRs have been conducted to compare the effects of meals after an overnight fast of varying macronutrient or micronutrient compositions, or different energy densities, on DIT. This highlights the need for a SR to be conducted in this area to address the lack of cohesive evidence of the role of these meals on DIT. This is of particular interest because even small changes in DIT may have significant effects on body weight and/or body composition over the longer term. Specifically, it has been suggested that an imbalance of 10–20 kcal/day can result in 0.5–1 kg of weight gain annually [11].

The primary question in this paper is whether there is any difference in the effects on DIT of meals consumed after an overnight fast of varying energy intake or macronutrient composition. Secondary questions are whether there is any difference in the effects on DIT if the same meal content is consumed using different eating patterns, such as a bolus meal versus repeated snacking, the effects of fast versus slow consumption of food, and whether food palatability of this meal has an effect on DIT. The outcomes of this SR will assist in better understanding the role of meal or snack consumption after an overnight fast on DIT and help to inform further research on the potential role of breakfast in health, as well as obesity prevention, treatment, and management.

## 2. Materials and Methods

The SR protocol was published in ‘Prospero’ (CRD42014009030). Methodological decisions about the review process were made a priori.

### 2.1. Search

With the assistance of a research librarian, four databases were searched: Cochrane (from 1992 to 14 May 2014), Cinahl (from 1937 to 14 May 2014), Embase (from 1947 to 14 May 2014), and Medline (from 1946 to 14 May 2014), and were then updated on the 23 February 2016 in order to capture the studies published between 14 May 2014 and 23 February 2016. This search used the following keywords: breakfast, morning meal, diet induced thermogenesis, thermogenesis, meal induced thermogenesis, thermic effect of food, resting metabolic rate, postprandial or post prandial metabolism, postprandial or post prandial metabolic rate, postprandial or post prandial energy expenditure, resting energy expenditure, postabsorptive or post absorptive energy expenditure, postabsorptive or post absorptive metabolic rate, basal metabolism, basal metabolic rate, and metabolic rate. For Embase, Medline, and Cinahl, limits were applied to include only human studies, those in English, and those conducted in adults. The Cochrane database did not allow these limits, however the word ‘adults’ was added as a keyword in order to limit the search to studies performed in adults. On the 23 February, this SR search was also expanded by the substitution of the keyword ‘morning meal’ by the keyword ‘meal’, in order to find studies that administered a meal after an overnight fast (breakfast) but did not use the terms morning meal or breakfast in the article. Also the keywords ‘thermic’ and ‘thermogenic’ were added to the search to ensure studies using slightly different language were not missed.

### 2.2. Eligibility Criteria

#### 2.2.1. Inclusion and Exclusion Criteria

For this review, only studies designated as level A evidence (randomized controlled trials (RCTs) and randomized crossover trials), as defined by the Academy of Nutrition and Dietetics, and with two or more eating events for comparison, were included. Studies were included if they provided a snack or a meal in the morning after participants fasted overnight. Studies were excluded if they provided infusions, injections, or capsules with the meal (e.g., saline or drug infusion, drug or placebo capsules). Interventions consisting of meals administered as enteral or intranasal or intra-gastric infusion or consisting of supplements instead of meals (e.g., protein or fat or sugar emulsions) or of meals supplemented with other components (e.g., addition of cellulose or pectin) including stimulants (e.g., caffeine, green tea, chilli, capsaicin, alcohol) were excluded. In these studies, the control meal (e.g., oral feeding or meal without stimulants) data were extracted if provided. When studies provided additional non-dietary interventions (e.g., exercise, sleep interventions), only data from the first meal consumed after an overnight fast intervention of the controlled arm were extracted.

Studies were included if they were published in English with male and/or female adult (≥18 years old) human participants. Data from populations such as children, adolescents, athletes or exercise-trained groups, patients with chronic or acute disease, obese individuals, pregnant or lactating women, or smokers were excluded. Studies with mixed populations of healthy weight, overweight, and obese populations were included; however, studies targeting only obese subjects or a mix of overweight and/or obese participants were excluded. Studies were also excluded when the majority of the participants were obese leading to a mean or median body mass index (BMI) ≥30 kg/m^2^. When studies compared specific populations (e.g., obese, pregnant women, athletes, smokers) to a control group, only data from the control group were included. The original search included all study designs, however, only RCT or randomized cross over designs were included in the analyses for this paper. Articles were excluded if they were expert opinion papers or if they described animal, in vitro, or in vivo experimental studies.

#### 2.2.2. Outcome Measures (Dependent Variables)

Diet induced thermogenesis measured by indirect calorimetry was the main outcome measure. Other outcomes of interest were indirect calorimetry fasting RMR and postprandial energy expenditure.

#### 2.2.3. First Meal Consumed after an Overnight Fast (Independent Variables)

The intervention was the first snack or meal of the day consumed in the morning after an overnight fast. Macronutrient compositions were described as percentages of the energy content of the meal. Energy was expressed in kJ.

#### 2.2.4. Systematic Review Process

Titles and abstracts were assessed for full text retrieval (A.Q.). Full text articles were assessed against the inclusion and exclusion criteria by two independent reviewers (A.Q. and A.P.). The quality criteria checklist for primary research of the Academy of Nutrition and Dietetics was used to assess the quality of the included studies by two independent reviewers (A.Q. and L.M.-W. or A.P.). The quality criteria tool assessed the studies for relevance and validity of the selected publications. A study was deemed positive if it met all the priority criteria, at least one of the validity criteria, and all of the relevance questions. A neutral rating indicated that most of the validity criteria were met but the study may not have met one or more of the priority criteria and/or one or more of the relevance questions. A study was rated as negative if six or more of the validity and/or priority criteria were rated negative. Any discrepancies between reviewers at the full text and quality stage were assessed by a fourth reviewer (R.C.) until a consensus decision was reached.

#### 2.2.5. Data Extraction

The relevant data from the studies were extracted into tables (A.Q.) and evaluated for completeness (A.P., R.C., L.M.-W.). The following information was extracted: study design, significance, inclusion and exclusion criteria, country location, sample size, participant characteristics (intervention and comparator groups), recruitment, blinding used, intervention, statistical analysis, timing of measurements, dependent and independent variables, co-variates, length of follow up and results (key findings and other findings), and author conclusions.

#### 2.2.6. Participant Characteristics

Participant characteristics (age, gender, BMI, fat mass (FM), and fat free mass (FFM)) were extracted when provided or calculated from the data provided. BMI was calculated using the WHO criteria as illustrated in Supplementary Materials Table S1. FM and FFM were expressed as % of total body weight or in kg. If only individual participant data were provided, the mean, standard deviation (SD), and standard error (SE) were calculated with the formula described in Supplementary Materials Table S1. The percentage of males in the sample was calculated (100% indicated that only males were recruited and 0% only females).

#### 2.2.7. Characteristics of the Meals

The energy content of the meals was expressed in kJ. The conversion factor of 4.184 was used to convert kcal to kJ. When studies provided the macronutrient composition of meals only in grams, it was converted from g to % of energy using the two formulas described in Supplementary Materials Table S1.

#### 2.2.8. Outcome Characteristics

RMR, also known as Resting Energy Expenditure (REE), is defined as the quantity of energy used to maintain physiological function under resting conditions. DIT, also called the thermic effect of food, postprandial energy expenditure above baseline, or meal-induced thermogenesis, is defined as the increase in RMR as a result of the consumption of food or a meal [12,13]. DIT data were extracted in kJ and/or as the percentage of energy content of the meal (ECM) and/or as the percentage increase above baseline (AB) RMR. When the studies provided only the total postprandial energy expenditure and the RMR, the mean DIT was obtained from the difference of the total postprandial energy expenditure in kJ and the RMR in kJ for the same measure of time. DIT expressed in kJ or as percentage of energy content was divided by the number of hours that DIT was measured, or multiplied by 60 if it was provided in kJ/minute, in order to provide values as kJ per hour or percentage per hour. The formulas described in Supplementary Materials Table S1 were used to convert DIT from one unit of measurement to another when not provided by the authors.

#### 2.2.9. Meta-Regressions

The main outcome variable used in the meta-regression was DIT in kJ/h. Mixed model meta-regression was used to investigate the relationship between energy intake (kJ) after an overnight fast and DIT (kJ/h). The first model conducted was a univariate analysis, which only included DIT (kJ/h) and kJ intake. The second model also included four confounding factors (percentage of males, age, BMI and hours of DIT measurement). These meta-regression models were conducted using Stata/IC 13.1 (StataCorp LP, College Station, TX, USA) and with consultant statistical support.

#### 2.9.10. Meta-Analyses

Fixed model meta-analyses were conducted in Review Manager (RevMan) to determine the mean difference in DIT (kJ/h) of pairs of comparisons. These meta-analyses were conducted with consultant statistical support.

## 3. Results

**Figure 1:**
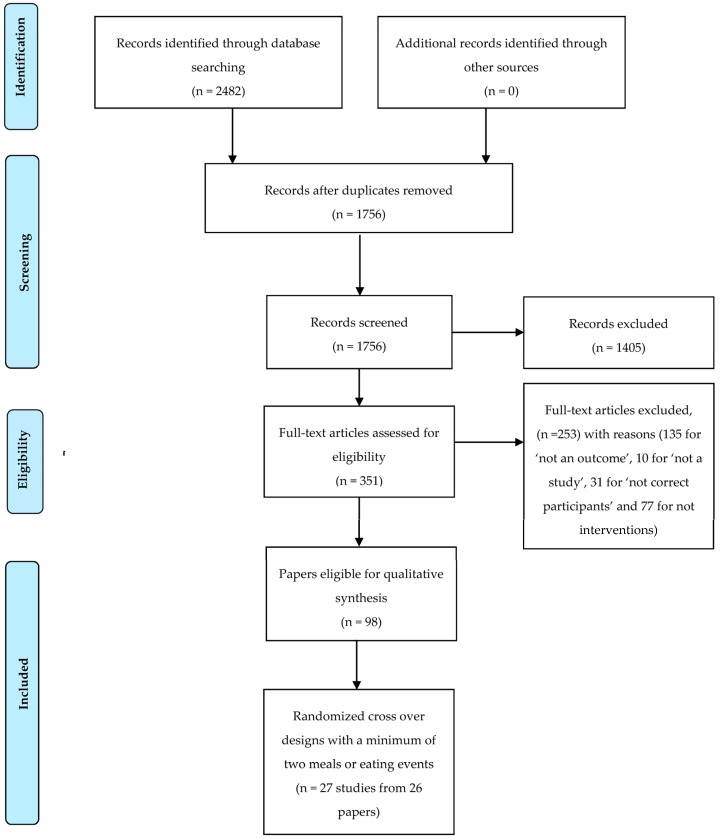
PRISMA Flow diagram [40] systematic search and review process.

A total of 2482 papers were identified from the four databases searched; 1756 papers remained after duplicates were removed and 351 full text articles were reviewed (Figure 1). Only 27 Level A evidence studies from 26 papers (one paper described two studies [14]) were relevant to answer the review questions for this paper. Of the 26 papers, four were rated positive [15,16,17,18], none were rated negative, and the remaining 22 papers were rated neutral.

Table 1 summarizes the 27 studies for participants’ characteristics and study protocols. Table 2 summarizes the interventions and outcomes of the studies. Nine studies were conducted in the USA [19,20,21,22,23,24,25,26,27], five in Japan [14,28,29,30], four in the UK [17,31,32,33], two in Australia [18,34], two in France [35,36], two in Denmark [15,37], one in Germany [38], one in Spain [39], and one in the Netherlands [16].The majority of the studies were not blinded, three were double blinded [14,37], and two studies were single blinded [18,24]. One study provided intervention meals for two weeks for each arm [36] whereas all other studies provided only one day interventions.

### 3.1. Participant Characteristics

A total of 350 participants were included. The participants’ characteristics are described in Table 1. The participants mean ages ranged from 20 to 69.4 years. Mean BMI ranged from 18.1 to 27.8 kg/m^2^. FM was mostly expressed in % and it ranged from 11.1% to 31.4%. FFM was only described in kg and it ranged from 41.7 to 66.8 kg. The sample size ranged from a minimum of four to a maximum of 29. The majority of the studies had a sample size <20; only four studies had ≥20 participants [24,33,35,39]. Fifteen studies had only males [14,18,19,21,22,23,25,28,30,33,34,35,36,37,39], eight studies had only females [14,20,24,27,29,31,32,38], and four studies had a mix of males and females [15,16,17,26].

### 3.2. Interventions

All studies required participants to attend the research setting in the morning after an overnight fast. The majority of the studies required arrival at the research center after a fasting period ranging from 10 to 14 h [15,18,20,22,23,24,25,26,27,28,29,31,33,34,35,37,38]. Many studies also required refraining from any exercise/physical activity or vigorous exercise either from the evening/dinner before [24,28,30,34], or from the day before [17,19,20,29,35], or an even longer period of time (36 h to 3 days) [15,18,21,22,23,25,37]. Fasting RMR was then measured and the meal administered. The interventions differed in energy intake and macronutrient composition between studies. Energy intakes for meals ranged from 418 to 6276 kJ. Carbohydrate (CHO) ranged from 0% to 90.4%, protein from 1.3% to 34.0%, and fat from 1.0% to 78.8% of energy intake. DIT was measured after the meal was consumed.

### 3.3. Outcomes

The mean fasting RMR measured before administering the meals ranged from 191.6 to 375.3 kJ/h. The majority of studies measured DIT periodically rather than continuously over two [34], three [22,23,29], three and a half [24,30], four [35,36], five [15,18,20,26,27,31], or six hours [14,17,19,26,31,38]; only eight studies measured it continuously over one and a half [28], three [33], three and a half [16], four [25], five [15,37,39], or six [21] hours. Therefore, the duration of the DIT measurement period ranged from one and a half to six hours. The majority of studies measured DIT for five [15,18,20,26,27,31,37,39] or six hours [14,17,19,21,26,32,38]. Mean DIT ranged from 4.5 to 99.4 kJ/h and from 0.77 to 4.3%/h (ECM), or from 1.3% to 41% above baseline (AB).

### 3.4. Comparison and Meta-Regression of the Effects of Higher and Lower Energy Intakes on DIT

Five studies [21,22,23,31,36] with the primary aim of comparing the effects of meals with different energy intakes on DIT were identified. Three studies [23,31,36] found an increased DIT when a higher energy intake was consumed, although only one indicated statistical significance [31]. Kinabo and Durbin [31] compared high CHO, low fat meals at two energy intake levels: 2520 kJ and 5040 kJ, and low CHO, high fat meals at the same two energy intake levels. This study found that a higher energy intake was associated with a significantly (*p* < 0.001) higher DIT, regardless of dietary composition. Higher energy intake (5040 kJ) resulted in a similar DIT for the high CHO, low fat (71.2 (15.5) kJ/h) and low CHO, high fat (68 (12.4) kJ/h) meals, and this was higher than the DIT for the lower energy intake (2520 kJ) high CHO, low fat (45.6 (9.3) kJ/h) and low CHO, high fat (45.6 (10.8) kJ/h) meals [31].

Hill et al. [23] compared DIT following three meals of 2092 kJ, 4184 kJ, and 6276 kJ [23]. DIT was higher with higher energy intake: 2092 kJ meal DIT <10% above baseline RMR; 4184 kJ meal DIT 21% above baseline RMR, and 6276 kJ meal DIT 33.5% above baseline RMR, no *p* value provided [23].

Martin et al. [36] compared two weeks of low energy, moderate fat meals (418 kJ) to two weeks of high energy, low fat meals (2929 kJ) and found a higher DIT after the high energy, low fat meals (low energy, moderate fat meals 4.5 (1.4) kJ/h; high energy, low fat meals 35.6 (2.6) kJ/h; no *p* value provided [36]).

Bennet et al. [21] compared a high fat meal (kJ not provided) to a normal fat meal (kJ not provided). The high fat meal was 1881 kJ higher due to the addition of 50 g of fat compared to the normal fat meal. This study did not find any significant differences in DIT (high fat meal 1.2 (0.40) %/h, normal fat meal 1.3 (0.3) %/h; *p* > 0.05 for %/6 h ECM for all participants, including some trained individuals, for the statistical tests) [21].

Segal et al. [22] compared consuming a meal with a fixed energy intake (3013 kJ) to a meal providing 35% of each individual’s 24 h RMR (caloric intake varying between participants, on average 2889 kJ intake) [22]. This study did not find any significant difference in DIT (fixed: 96.3 (17.6) kJ/h; 35% RMR: 89.3 (17.6) kJ/h, *p* > 0.05 for %/3 h ECM) but there was little difference in the energy intakes between the two meals [22].

In order to further resolve the effect of energy intake on DIT, mixed model meta-regression analyses were undertaken to investigate more broadly the relationship between energy intake (kJ) after an overnight fast and DIT (kJ/h). Two models were produced: the first one included only energy intake (kJ) and the outcome variable DIT (kJ/h); the second model also included four confounding factors (percentage of males, BMI, age, and hours of DIT measurement).

**Figure 2:**
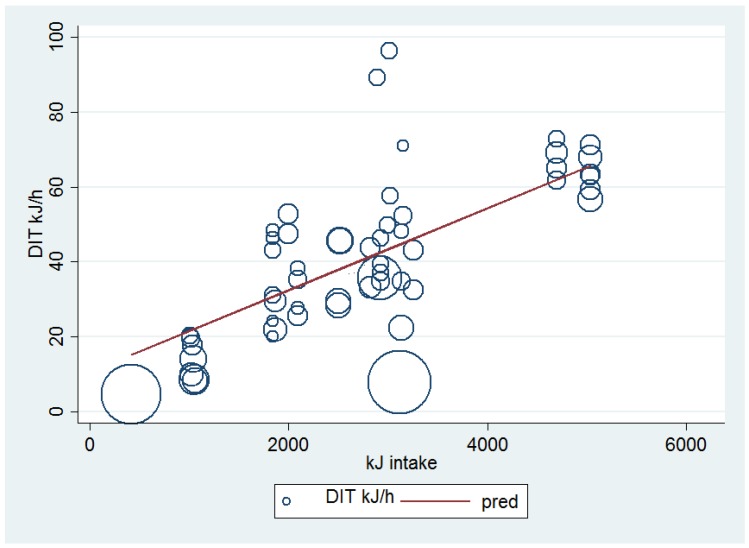
Mixed Model Meta Regression: univariate association between energy intake (kJ) and DIT (kJ/h) (Model 1). The Figure is composed of circles and a regression prediction line (in red) representing the outcome (DIT); each circle represents the value of DIT (kJ/h) for an arm of a study, and the size of the circle is inversely proportional to the standard error (SE) of the study. The influence of each study on the model depends on the size of the SE. Specifically, a study arm with a large SE is represented in the figure by a small circle, which means that this study arm had a small influence on the model whereas a study arm with a small SE is represented by a large circle, which means that this study arm had a large influence on the model.

Figure 2 represents Model 1 (coefficient 0.011, standard error 0.0013, *p* < 0.001, 95% confidence interval, (CI) 0.0083; 0.014) conducted for 19 studies [14,16,17,18,19,22,24,25,27,30,31,32,33,34,35,36,37,38] with a total of 54 treatment arms. Eight studies could not be included in the meta-analyses because they had missing values for one or more of the variables investigated in the model. This model shows that DIT (kJ) increases significantly (*p* < 0.001) when the kJ content of meals increases, although this increase is of a small magnitude (coefficient 0.011). This model predicts that for every 100 kJ increase in energy intake, DIT increases by 1.1 kJ/h.

Model 2, adjusted for percentage of males, BMI, age, and hours of DIT measurement, also predicted a small but significant increase in DIT for every kJ intake (coefficient 0.012, standard error 0.0013, *p* < 0.001; CI: 0.0091; 0.014). This model predicts that for every 100 kJ increase in energy intake, DIT increases by 1.2 kJ/h. In this model, 16 studies were included with a total of 48 arms. Three studies included in model 1 were not included in model 2 because they had missing values for one or more of the variables investigated [16,25,34]. DIT accounted for 47.4% of the variance in Model 1 and 70.6% of the variance in Model 2.

### 3.5. Influence of Macronutrient Composition on DIT

Six studies [15,20,25,30,31,33] compared meals differing in macronutrient composition (fat vs. CHO and/or vs. protein). Five of these papers compared consuming a meal high in CHO with a meal high in fat. Nagai et al. [30] reported a higher DIT with a high CHO meal (3255 (306.5) kJ) compared to an isocaloric meal high in fat (3255 (306.5) kJ). DIT was 43.1 (13.7) kJ/h for the high CHO meal and 32.6 (14.1) kJ/h for the high fat meal, *p* < 0.05 for %/3.5 h ECM [30]. Blundell et al. [33] provided isocaloric comparisons (both meals contained 2092 kJ) and found a statistically significant effect on DIT (high CHO milkshake: habitually high fat consumers 38.2 (26.0) kJ/h and habitually low fat consumers 35.2 (15.6) kJ/h; high fat milkshake: habitually high fat consumers 27.5 (28.9) kJ/h and habitually low fat consumers 25.6 (14.5) kJ/h, *p* < 0.05 for kJ/day) [33]. The other two studies provided meals with only small differences in energy content (high CHO meal 2068 kJ and high fat meal 2093 kJ) [20]; high CHO meal 3021 (1194.0) kJ and moderate fat meal 2996 (1167.4) kJ) [25]), and DIT was as follows: high CHO meal 54.6 kJ/h, high fat meal 27.8 kJ/h [20]; high CHO meal 57.8 (19.1) kJ/h and moderate fat meal 49.8 (21.6) kJ/h [25]. No *p* values were provided for these comparisons; therefore, it is not known if these comparisons were statistically significantly different [20,25].

One study provided isocaloric comparisons and found no significant effect on DIT between high CHO, low fat meals and low CHO, high fat meals [31]. This study [31], which was described in Section 3.4 (higher energy vs. lower energy intake), provided the same group of subjects with high CHO, low fat meals of two different energy contents (2510 kJ and 5040 kJ), as well as low CHO, high fat meals of two different energy contents (2520 kJ and 5040 kJ) [31]. The DIT data were as follows: 5040 kJ high CHO, low fat meal 71.2 (15.5) kJ/h and 2520 kJ high CHO, low fat meal 45.6 (9.3) kJ/h vs. 5040 kJ low CHO, high fat meal 68 (12.4) kJ/h and 2520 kJ low CHO, high fat meal 45.6 (10.8) kJ/h, *p* > 0.05 for kJ/5 h comparing high CHO, low fat meals with low CHO, high fat meals [31].

Additionally, only one study [15] compared consuming isocaloric meals rich in protein vs. fat vs. CHO in participants of the same sex (females consumed 2500 kJ and males 3000 kJ). The high CHO and fat meals had the same DIT, whereas the high protein meal had a higher DIT (CHO meal 39.2 kJ/h, fat meal 39.2 kJ/h, and protein meal: 45.9 kJ/h, *p* < 0.01 for %/5 h ECM comparing four meals (an alcohol meal was excluded for the purpose of this SR)) [15].

One study [38] investigated the effect of consuming an adequate level of protein (3131 kJ) with a low level of protein (3114 kJ) in meals with similar energy contents. This study found a higher DIT when an adequate level of protein was consumed compared to a lower level (adequate protein meal 22.4 (5.7) kJ/h; low protein meal 7.8 (1.0) kJ/h, *p* = 0.001 for kJ/6 h and %/6 h) [38].

Riggs et al. [24] undertook isocaloric comparisons of meals differing in the amount of fat provided and found a higher DIT after consuming a moderate fat meal (1841 kJ) than an isocaloric low fat meal (1841 kJ), where both meals were high in protein, among normal weight participants (*p* < 0.005 for in %ECM) but not in overweight or underweight participants [24]. The DIT results were as follows; normal weight: moderate fat meal 43.1 (19.2) kJ/h vs. low fat meal or 31.0 (19.2) kJ/h; overweight: moderate fat meal 48.4 (20.0) kJ/h vs. low fat meal 46.3 (18.8) kJ/h; underweight: moderate fat meal 20.0 (16.4) kJ/h or vs. low fat meal 24.2 (15.6) kJ/h [24].

#### 3.5.1. Long Chain Triglycerides vs. Medium Chain Triglycerides

Three studies [14,17] compared meals containing medium chain triglycerides (MCT) with long chain triglycerides (LCT) and all found a statistically higher DIT with meals containing MCT rather than meals containing LCT. Clegg et al. [17] provided two meals of the same energy content and macronutrient profiles but containing either MCT (20 g) or LCT (18.4 g) (1863 kJ) [17] and found a significantly higher DIT with the MCT meal (MCT: 29.4 (8.4) kJ/h vs. LCT: 21.9 (7.9) kJ/h, *p* < 0.005 for %/6 h ECM) [17].

Kasai et al. [14] conducted two studies comparing the effects of MCT vs. LCT. In study 1, three meals were administered (5 g of MCT and 5 g of LCT (1029 kJ) meal, 10 g MCT meal (1013 kJ), 10 g LCT meal (1046 kJ) [14]. DIT was significantly increased when MCT meals were consumed vs. LCT (10 g MCT meal 19.5 (14.1) kJ/h vs. 10 g LCT meal 8.4 (4.6) kJ/h, *p* < 0.01 for % ECM). Furthermore, DIT was significantly higher for the meal with both MCT and LCT than the one containing only LCT (5 g MCT, 5 g LCT meal 17.7 (10.8) kJ/h vs. 10 g LCT meal 8.4 (4.6) kJ/h, *p* < 0.01 for %/6 h ECM) [14].

In Study 2 [14], four meals were administered containing: mayonnaise with 5 g MCT (1042 kJ), mayonnaise with 5 g LCT (1059 kJ), margarine with 5 g MCT (1004 kJ), or margarine with 5 g LCT (1020 kJ) [14]. This study found a significantly higher DIT with meals containing MCT as opposed to LCT (meal with mayonnaise and MCT: 14.0 (5.7) kJ/h vs. meal with mayonnaise and LCT: 8.2 (6.4) kJ/h, *p* < 0.05 for % ECM, meal with margarine and MCT 20.3 (15.7) kJ/h vs. meal with margarine and LCT 9.8 (8.2) kJ/h, *p* < 0.05 for %/6 h ECM [14].

**Figure 3:**
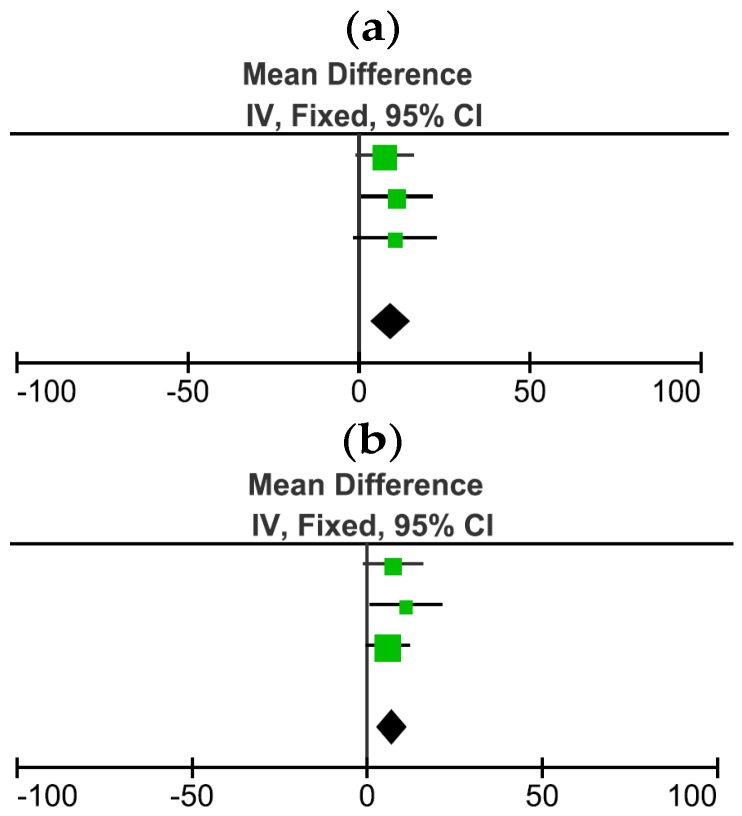
(**a**) Meta-analysis with fixed effect of the mean differences in DIT between MCT and LCT with the margarine trials of Kasai et al. Studies included in this meta-analysis are represented in the figure by symbols (green squares) and they are illustrated in the following order: Clegg et al. [17]-46.0% weight; Kasai et al. [14]-study 1-31.6% weight; Kasai et al. [14]-study 2 margarine trial-22.4% weight [14]; (**b**) Meta-analysis with fixed effect of the mean differences in DIT between MCT and LCT with the mayonnaise trials of Kasai et al. Studies included in this meta-analysis are represented in the figure by symbols (green squares) and they are illustrated in the following order: Clegg et al. [17]-28.6% weight; Kasai et al. [14]-study 1-19.6% weight; Kasai et al. [14]-study 2-mayonnaise trial-51.8% weight [14]. The % contribution of each study to the outcome is indicated as % weight.

A fixed model meta-analysis was conducted with these three studies [14,17] to compare DIT (kJ/h) for the MCT vs. LCT arms. Because Kasai et al. (2002) in study 2 [14] administered two interventions for MCT (margarine or mayonnaise) and two interventions for LCT (margarine or mayonnaise) to the same people, two forest plots are presented (one with only the margarine interventions and the other one with only the mayonnaise intervention arms). This avoids the effects of repetition of the same participants in both the margarine and mayonnaise studies. Both analyses found a significantly higher DIT when MCT was consumed compared to LCT (*p* = 0.002; Figure 3a,b). For both models the heterogeneity is 0% with chi^2^ = 0.3 and *p* = 0.9 (Figure 3a), and chi^2^ = 0.7 and *p* = 0.7 (Figure 3b). The total sample size is 23 (for Figure 3a) or 22 (for Figure 3b) for each group of comparisons with the same people repeated for both interventions.

#### 3.5.2. Monounsaturated Fat vs. Polyunsaturated Fat

Two studies [18,39] compared meals containing monounsaturated fatty acids (MUFA), polyunsaturated fatty acids (PUFA), or saturated fatty acids (SFA). Casas-Agustench et al. [39] found a significantly higher DIT after the consumption of meals containing PUFA (mean (95% CI) 2655 (2510–2799) kJ) or MUFA (mean (95% CI) 2608 (2428–2788) kJ) compared to the one containing SFA (mean (95% CI) 2599 (2421–2278) kJ). The DIT as mean (95% CI) was: PUFA meal 37.2 (29.5–44.8) kJ/h, MUFA meal 36.8 (30.5–43.0) kJ/h, and SFA meal 30.0 (24.2–35.8) kJ/h, *p* < 0.05 for kJ/5 h amongst the three interventions [39].

Contrary to this finding, the study by Piers et al. [18] found no significant difference in DIT (SFA meal 29.6 (10) kJ/h vs. MUFA meal 28.4 (10) kJ/h, *p* > 0.05 for %/5 h ECM and *p* > 0.05 for kJ/5 h between meals containing MUFA or SFA (both meals: 2500 kJ) [18].

#### 3.5.3. Structure of Fats

Bendixen et al. [37] compared consuming meals with either a conventional fat (sunflower oil) or a chemically structured fat (rapeseed oil and octanoic acid by esterification with sodium methoxide) or a lipase-structured fat (rapeseed oil and octanoic acid by esterification with lipoxime IM) or a physically mixed fat (blending rapeseed oil and trioctanoate) [37]. The mean energy content of these four meals was 4698 (550.2) kJ [37]. This paper found a significant effect of fat structure on DIT with the highest DIT associated with the meal containing a chemically structured fat and the lowest with the meal having the conventional fat (conventional fat meal 61.8 (15.2) kJ/h, chemically structured fat meal 72.8 (19.0) kJ/h, lipase-structured fat meal 69.2 (11.4) kJ/h, and physically mixed fat meal 65 (13.9) kJ/h, *p* = 0.005 for kJ/5 h) [37].

### 3.6. Processed vs. Unprocessed Food

Only one study [26] compared consuming two meals with different levels of processing. One meal was composed of whole food (multi-grain bread and cheddar cheese either as one and a half sandwiches or two sandwiches) and the other was composed of processed foods (white bread and a processed cheese either as one and a half sandwiches or two sandwiches) [26]. Subjects could choose to consume either one and a half sandwiches (2520 kJ) or two sandwiches (3360 kJ), and this choice was kept constant for both trials, thus the two trials were isocaloric for the same participant. There was a highly significant increase in DIT after consuming the whole food meal compared to the more processed meal (whole food meal: 99.4 (40.7) kJ/h; processed meal: 63.9 (35.6) kJ/h, *p* < 0.001 for total kJ and *p* < 0.01 for total % ECM [26]).

### 3.7. One Bolus Event vs. Isocaloric Smaller Frequent Meals

Four studies [27,32,34,35] compared administering a meal as a bolus event versus splitting the same meal into two [32], three [34], four [35] or six [27] smaller equal meals or snacks to be consumed throughout the morning. The time between multiple meals was 180 min [32], 60 min [35], or 30 min [27,34]. Kinabo and Durbin [32] compared two eating patterns using two different meal compositions: high CHO, low fat and low CHO, high fat. All four studies had the same participants perform both interventions (total *n* = 55). The energy density was as follows: 5040 kJ or 2510 kJ × 2 either as high CHO and low fat meal or low CHO and high fat meal [32]; 3150 or 1050 kJ × 3 [34]; 2823.4 kJ or 705.8 KJ × 4 meals [35]; and 3138 kJ or 523 kJ × 6 [27].

Two studies [32,34] found no significant difference in DIT between the bolus and the isocaloric smaller frequent meals event (high CHO, low fat meal as bolus 62.8 (13.2) kJ/h vs. smaller frequent meals event 63.5 (11.7) kJ/h, *p* > 0.05 for kJ/6 h; low CHO, high fat meal as bolus 59.3 (11.5) kJ/h vs. smaller frequent meals event 56.7 (8.0) kJ/h, *p* > 0.05 for kJ/6 h [32]; bolus 71 (31.5) kJ/h vs. smaller frequent meals 52.3 (15.3) kJ/h, *p* > 0.05 for kJ/2 h [34]).

The other two studies found a significantly higher DIT when the meals were consumed as a bolus event compared to smaller frequent meal events: bolus 43.8 (18.4) kJ/h vs. smaller frequent meals 33.2 (15.5) kJ/h, *p* < 0.05 for %/4 h ECM [35]; bolus 48.2 (16.93) kJ/h vs. smaller frequent meals 34.9 (12.3) kJ/h, *p* < 0.05 for kJ/5 h [27]).

**Figure 4:**
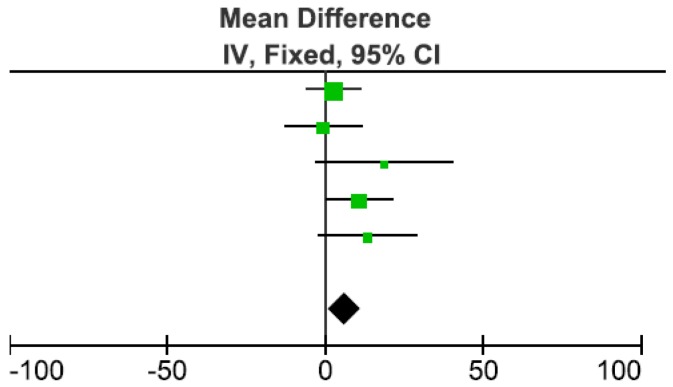
Meta-analysis: mean differences in DIT between bolus vs. smaller frequent meals event (such as snacking). Studies included in this meta-analysis are represented in the figure by symbols (green squares) and they are illustrated in the following order: Kinabo and Durbin et al. [32]-Paper B low CHO, high fat meal-37.6% Weight; Kinabo and Durbin et al. [32]-Paper B high CHO, low fat meal-19.0% weight; Vaz et al. [34]-6.0% weight; Allirot et al. [35]-25.5% weight; Tai et al. [27]-11.8% weight. Weight refers to amount of influence that the study exerts on the meta-analyses. The % contribution of each study to the outcome is indicated as % weight.

In order to clarify these discrepancies, a meta-analysis of the mean differences between bolus and smaller frequent meal event trials with fixed effects was conducted in RevMan to find the overall effect on DIT [32]. For these analyses, DIT was compared in kJ/h in order to standardize the units between studies. The forest plot shows (Figure 4) the mean of the difference between bolus and smaller frequent meal event trials for each study. The overall mean of the difference is positive, which means that the DIT was lower in the smaller frequent meals event trials compared to the bolus trial. This overall effect on DIT was significant (*p* = 0.02). The heterogeneity was 14%, chi^2^ = 4.6 and *p* = 0.3.

### 3.8. Fast vs. Slow/Normal Meal Consumption

Two studies [28,29] compared consuming the same isocaloric meal quickly or more slowly. One study [28] compared eating a meal (1255.2 kJ) as fast as possible to a meal chewed as many times as possible until no lumps remained before swallowing. The other study [29] compared eating a meal (1464 kJ) in 15 min compared to 5 min. Both studies found a higher DIT when the meal was consumed by slower eating compared to fast eating (slower eating 502.1 (234.4) kJ/kg/h vs. fast eating 19.5 (142.2) kJ/kg/h, *p* < 0.05 for kcal/kg/90 min [28]; slower eating 41.9 (14.6) kJ/kg/h vs. fast eating 31.6 (15) kJ/kg/h, *p* > 0.05 for cal/kg/180 min) [29]), although only one of the studies reached statistical significance [28].

### 3.9. Palatable vs. Unpalatable

Two studies [16,19] compared consuming palatable vs. unpalatable isocaloric meals (2930 kJ [19]; 2000 kJ [16]) on DIT. There was no significant difference in DIT between these two approaches indicating palatability did not influence DIT. In the first study [19], the effects of palatability were examined in both young and old participants (old participants: palatable meal 37.0 (15.9) kJ/h vs. unpalatable meal 46.4 (18.4) kJ/h; young participants: palatable breakfast 34.7 (13.6) kJ/h vs. unpalatable meal 39.5 (17.0) kJ/h, *p* > 0.05 for %/3 h ECM). In the second study there was also no difference in DIT with palatability (palatable meal 47.3 (14.2) kJ/h vs. unpalatable meal 52.9 (13.3) kJ/h, *p* > 0.05 for kJ/3/5 h and %/3.5 h [16]).

## 4. Discussion

This review investigated the effects of meals consumed after an overnight fast that differed in energy content or macronutrient composition on DIT, as well as the effects of consuming the same meal as a single event or multiple small meals or snacks. Studies comparing the effects of differing energy intakes supported a conclusion that a higher energy intake resulted in a higher DIT. This finding was further supported by two meta-regressions (one unadjusted and one adjusted for confounding factors), which found that for every 100 kJ increase in energy intake, DIT increased by 1.1 (unadjusted) or 1.2 (adjusted) kJ/h. A number of studies compared the effects of meals differing in macronutrient composition. One study found that a meal high in protein resulted in a higher DIT than meals high in CHO or fat, and a number of studies suggested that a meal high in CHO resulted in a higher DIT than a meal high in fat. Medium chain triglyceride meals produced a higher DIT than long chain triglycerides, the effects of mono- and polyunsaturated fats compared to saturated fats were unclear, fat structure (e.g., sunflower oil compared to a chemically structured fat) influenced DIT, and the fat content of a meal had inconsistent effects on DIT. The DIT of meals consumed as two or three small meals did not differ to the DIT of the same meal consumed as a single meal, whereas meals consumed as four to six small meals had a lower DIT compared to the same meals consumed as a single meal. Together these findings indicate that meals consumed after an overnight fast result in a DIT and the magnitude of this DIT is influenced by the energy content, the macronutrient composition, and the eating pattern of the meal.

Five studies investigated the effects of consuming different energy intakes on DIT as a primary outcome [21,22,23,31,36]. The study with the largest sample size found a significant increase in DIT with a higher energy intake [31]. Two studies with much smaller sample sizes reported trends of a higher DIT with a higher energy intake [23,36]. Two other studies [21,22] found no effect on DIT but the small sample sizes (eight and 11) could have impacted these findings. Additionally, one of these two studies provided little difference in energy intake between the two meals consumed [22]. The meta-regressions subsequently undertaken to examine the effect of energy intake on DIT across a much larger range of studies clearly support a conclusion that the energy content of meals consumed after an overnight fast influences DIT. Both the unadjusted meta-regression and the one adjusted for four confounding factors (percentage of males, age, BMI, and duration of DIT measurement) found similar significant relationships between a higher energy intake and a higher DIT. The magnitude of the increase in DIT was very small (1.1 or 1.2 kJ/h increase with each 100 kJ increase in energy intake), and whether this increase is clinically meaningful may depend on the magnitude of the energy content of the meal. These findings are consistent with the conclusion of Westerterp that energy intake is a predictor of DIT [41].

A number of studies compared the effects of meals consumed after an overnight fast differing in macronutrient composition. Five studies compared high fat vs. high CHO meals, and four of them found a higher thermogenic effect after the consumption of a high CHO meal compared to a high fat or moderate fat meal. The two studies that found significant effects had sample sizes of 24 males [33] and 14 males [30]. The other two studies, which were conducted with smaller sample sizes (12 males [25] and six females [20]), showed trends for a higher thermogenic effect of high CHO meals, but they did not provide *p* values and this limited their conclusions [20,25]. Furthermore, Thyfault et al. [25] compared a high CHO meal with a moderate fat, moderate CHO meal (45% fat) and therefore, the moderate CHO content could have confounded the findings. The one study that reported no significant difference for this high fat vs. high CHO comparison was conducted in 16 females [31]. Significant effects were found in the two studies conducted in males, whereas no significant effect was observed in the study in females, suggesting that males and females may respond differently following the consumption of CHO and fat meals. The differences in DIT between males and females may result from hormonal and/or body composition differences. Again, more research is needed to clarify these observations.

Two studies investigated the effects of protein on DIT; one found a significant increase in DIT after a high protein meal compared to high CHO or high fat meals in 19 participants of mixed gender [15]. The other study found a significant increase in DIT when a high protein meal was consumed compared to an adequate protein meal even though this was a small study of only six females [38]. Both studies suggest a high thermogenic effect of protein, however due to the limited numbers of studies, more research is needed to further investigate the thermogenic effect of protein in meals in both males and females, as gender was suggested to have an effect on the studies comparing high CHO vs. high fat meals [30,31,33].

A review by Tappy et al. [42] supports the higher thermogenic effect of protein compared to fat; this review reported DIT to be 0%–3% for fat, 5%–10% for CHO, and 20%–30% for proteins [42]. The different thermogenic effects of macronutrients are further reinforced by two studies comparing two diet interventions [43,44]. One study compared a high protein diet to an adequate protein diet for four days in 12 women and found a significantly higher DIT with the high protein diet (high protein diet 0.91 (0.25) MJ/day or 10.1 (2.7) % energy intake vs. high fat diet 0.69 (0.24) MJ/day or 7.6 (2.5) % energy intake, *p* < 0.05) [43]. Another study compared a high protein diet to a high fat diet for 36 h in eight women and found a significantly higher 24 h DIT with the high protein diet intervention as opposed to the high fat diet (high protein diet 1295 (240) kJ/day or 14.6 (2.9) % energy intake vs. high fat diet 931 (315) kJ/day or 10.5 (3.8) % energy intake, *p* = 0.02) [44]. Therefore, the findings of this SR regarding the role of meals differing in fat, CHO and protein composition on DIT are consistent with other studies investigating the effect of diets varying on macronutrients compositions on DIT. With regard to possible mechanisms of action of the higher thermogenic effect of protein, Westerterp-Plantenga [45] suggests that a higher protein diet may increase protein synthesis, which has a high energy cost, or if protein is oxidized the energy cost is higher than fat or CHO, and energy cost also varies with amino acid composition [45,46].

One study compared higher vs. lower levels of fat intake and found a significant increase in DIT following the consumption of a high fat meal compared to a low fat meal in female participants. This significant effect was only found in the 12 normal weight participants [24] and not in the six overweight or three underweight participants, however the small numbers of participants in the overweight and underweight groups would make finding effects in these groups difficult. Also, the different protein levels of the two meals could have impacted the findings of this study.

A number of studies compared the effects of different types of fats on DIT whereas no studies were identified that compared the effects of different types of proteins or carbohydrates on DIT. A significantly higher thermogenic effect was found when meals containing MCTs were compared to those containing LCTs; this finding was consistent in the three studies included [14,17]. The meta-analysis combining these three papers confirmed this significant increase in thermogenesis following the consumption of a meal with MCTs compared to LCTs (*p* < 0.005). Two hypotheses have been proposed regarding possible mechanisms by which the higher thermogenic effect of MCTs vs. LCTs might be achieved. One suggests an important role for the liver. MCTs are transported directly to the liver by the portal vein whereas LCTs are transported by the lymphatic system to peripheral tissues (adipose tissue and muscle) [47]. Also, LCTs need to bind to carnitine in order to pass through the mitochondrial membrane of the liver where B-oxidation occurs [47], whereas MCTs do not [48]. Therefore, MCTs being directly transported to the liver and that are easily able to pass through the mitochondrial membrane may be responsible for their higher DIT [49,50].The second hypothesis suggests a role for the sympathetic nervous system. Dullo et al. [51] found increased noradrenaline levels after MCT consumption and the authors suggested that sympathetic nervous system stimulation could therefore be responsible for the increase in energy expenditure of MCTs. Kasai et al. [14] has indicated that more research is needed to support this proposed mechanism.

Inconsistent results were found between two studies that compared different saturation of fat on DIT. The study which found a significantly higher DIT by PUFA and MUFA meals compared to a SFA meal [39] had a much larger sample size (29 participants) than the study which did not find a significant difference between MUFA and SFA meals (14 participants) [18]. More research is needed to clarify these findings. The effects of fat structure were also investigated. One study [37] found that fat structure, specifically meals containing a chemically structured fat, a lipase-structured fat, and physically mixed fat was associated with a significantly higher DIT compared to a conventional type of fat, and the highest DIT was associated with the consumption of the chemically structured fat. These findings are clearly preliminary and more studies are needed to confirm these observations.

One study examined the thermogenic effect of a less processed (e.g., whole grain bread) meal compared to a more processed meal [26] after an overnight fast and found a significantly higher DIT following consumption of the less processed meal. Although energy intake was consistent between trials in the same participant, the sample consisted of males and females, and there were two different caloric options within the study. It is unclear whether the choice of caloric options was controlled for in the analysis or whether there was a gender difference in the choice of caloric option. Furthermore, the macronutrient composition of these two meals differed and could have impacted the findings. Therefore, there is a need for further research comparing the effects of consuming more processed vs. less processed foods with the macronutrient content of the meals closely matched.

Four studies investigated the effects of consuming the same amount of calories and meal composition as a bolus event compared to a number of smaller meals during the morning. Two studies found a significant increase in DIT when the meal was consumed as one meal (bolus) instead of four [35] or six [27] smaller frequent meals. Two other studies did not find a significant difference in DIT between bolus and two [32] or three [34] smaller frequent meals regardless of macronutrient compositions [32,34]. The studies that did not find a significant difference provided less frequent meals for the snacking comparison, resulting in fewer meals with higher energy intake. The meta-analysis conducted on these four studies found that DIT was significantly higher when the meal was consumed as one bolus event. Together these results suggest that fewer larger meals result in a higher DIT than more frequent smaller meals.

Two studies [28,29] found a higher DIT following a meal eaten slowly compared to a meal eaten quickly, although these findings were only significant for one study [28]. The study that found a significant effect was conducted in ten males whereas the study that did not find a significant difference was conducted in nine females [29]. It is possible that gender is a factor influencing these results. Furthermore, these two studies suggest that the time that is spent on chewing the food may influence the magnitude of DIT; however more research is needed to clarify this observation and to compare the effects between males and females. Only two studies [16,19] have compared the effects of consuming a palatable versus an unpalatable meal on DIT in 19 males [19] or 12 participants (6 males and 6 females) [16]. Neither study found any significant differences in DIT. Although these findings suggest that palatability has no effect on DIT, the small number of studies limits the ability to draw any firm conclusions on this topic. Finally, it is important to note that no studies identified for this review have investigated the effects of differing micronutrients on DIT and this may potentially be another factor to influence DIT, which therefore warrants further investigation.

### 4.1. Strengths of This SR

This SR, including meta-analysis and meta-regression, is the first one to be conducted to investigate the effects of energy intake, macronutrient composition and eating events on DIT. This SR was able to identify and summarize the highest level of evidence available in this area and highlights where more research is needed in this field. The studies included were screened for quality/risk of bias and none of the studies had negative quality. Furthermore, the meta-regression was able to quantify the short-term effects of differing energy intakes after an overnight fast on DIT. In addition, the meta-analyses were able to quantify the influence of MCT vs. LCT and the role of consuming bolus eating events vs. smaller frequent meals in the morning on DIT. Considering the lack of evidence base regarding the role of meals consumed after an overnight fast on obesity prevention, this SR was able to provide evidence of the short-term effect of consuming different types of meals on DIT, which in the long term could play a significant role in obesity.

### 4.2. Limitations

There are a number of limitations regarding this SR. First, the included studies were very heterogeneous, differing in their research questions and the types of meals served after an overnight fast (as summarized in Table 1 and Table 2). This heterogeneity limited the meta-analyses that could be conducted and the confidence with which conclusions could be drawn. Furthermore, only meta-analyses with a minimum of three studies were included in this SR.

Secondly, the majority of studies investigating DIT are conducted after an overnight fast even if their primary aim is not to investigate the effect of breakfast per se. These studies met the criteria to be included because they administered meals/snacks (even if not a typical breakfast meal) after an overnight fast (definition of breakfast). Lacking are studies of the effects of meals representative of breakfast in specific cultures. Whether these representative breakfast meals would have a different effect on DIT is unclear.

Thirdly, the studies varied in the units of measurement used to report DIT, including kcal or kJ, % ECM, or % above baseline (AB), and this limited the direct comparability of the findings between the studies. In order to address this issue, whenever possible, DIT was converted into units of measurements that allowed direct comparisons (e.g., DIT kJ converted to % ECM or % AB). Also, the studies measured DIT for different lengths of time, ranging from one and a half to six hours, and the length of time that the DIT is measured affects the magnitude of DIT detected [12,13,52]. In order to account for this limitation, the data were transformed into kJ/h allowing the results to be compared among studies. Furthermore, this confounding factor was adjusted for in the meta-regression model.

There is conflicting evidence about the length of time that DIT needs to be measured to provide accurate results. Two papers have recommended measuring DIT for at least three hours. One conducted a study in ten participants and concluded that 3 h DIT measurements are sufficient as 76% of DIT is obtained during this period [52]. The other paper [12] analyzed data from six studies with a total of 103 subjects and also recommended measuring DIT for three hours, as they found that the majority of the DIT was measured by three and a half hours when either high or low energy intakes (ranging from 1.3 MJ to 2.6 MJ) were consumed and in both men and women [12]. Another study [13] conducted in 131 participants recommended measuring DIT up to six hours, as 3 h measurements underestimated the DIT response by 40% [13]. Therefore, it is possible that studies included in this review that measured durations of DIT shorter than six hours may have underestimated DIT. In addition, DIT was measured differently in the studies, as some measured DIT for short durations at regular intervals (interval ranges also differed among studies) whereas other studies measured it continuously, which may have affected the magnitude of DIT detected. Specifically, Piers et al. [53] found a significantly (*p* < 0.01) lower DIT when it was measured at intervals compared to continuously in the same five subjects [53]. Most of the studies included in this review measured DIT at intervals and therefore there is a risk that the DIT was underestimated.

The small sample size of the majority of the studies included is another limitation of this SR. Sample sizes ranged from four to 29 participants, which limits the statistical power and capacity to find small but possibly important effects, as well as limiting the generalizability of the findings. This makes similarity of study designs that can be included in meta-analyses and meta-regressions even more important.

The interventions provided in the studies included in the meta-regression on energy intake had substantial variations; for example, they had different macronutrient compositions, and in some instances the meals were administered differently (such as meals consumed as a bolus vs. smaller frequent meals). Furthermore, the studies included in the meta-analyses were few, and even then included some heterogeneity. For example, the studies included in the MCT and LCT meta-analysis had meals with different levels of LCT or MCT. Also, for the bolus vs. smaller frequent meals meta-analysis, the energy intakes differed between the studies included. Furthermore, the smaller frequent meals arm differed in the number of smaller meals provided between the studies (two, three, four and six). All these factors were not possible to be adjusted for and could have impacted the findings of the meta-regressions and meta-analyses.

Furthermore, the meta-analyses were conducted by considering the two groups for comparison as two different groups of people, even though in reality it was the same group of people repeated in cross-over designs. For this reason, the analyses conducted by RevMan used a more conservative approach, because it is more difficult to find statistical significance if two comparison groups consist of different people. However, the heterogeneity between studies is also expected to be underestimated for the same reason. Therefore, for the two meta-analyses conducted in this SR (MCT vs. LCT and meal vs. snacking), the heterogeneities are expected to be larger than the number provided and the *p* values are also expected to be even more significant (smaller) that the ones provided (in both cases they are already highly significant). The CIs are also believed to be smaller than the ones provided.

Also, in the findings of this SR, meta-regression and meta-analyses were based primarily on studies rated neutral for quality, thereby limiting the confidence with which conclusions could be drawn.

Finally, the included studies were almost entirely short-term (single meal interventions; only one study investigated the effects of 2 weeks of the same meal daily prior to measurement following a single meal) [36], therefore, whether the effects observed reflect, at least in part, the novelty of unfamiliar meals consumed after an overnight fast on DIT is unclear.

It was not possible to draw any conclusions about the effect of a routine breakfast on DIT. Therefore larger, longer-term experimental studies are needed to draw conclusions about these topics. Specifically, there is the need to investigate the long-term effects of regular consumption of low energy intake vs. high energy intake meals and meals varying in macronutrient composition on DIT, and whether these factors ultimately affect total daily energy intake and DIT or the weight of participants. There is also a need to compare the effects of these influences on DIT between regular breakfast eaters and skippers, as this was found to be an important factor to consider by a previous trial conducted in this area [54].

### 4.3. Recommendations

Most of the studies included in this SR were rated neutral instead of positive quality because they had not provided enough or clear information about the selection of participants, recruitment, or inclusion and exclusion criteria. Therefore, it is recommended for future studies in this area to provide more information about recruitment, inclusion and exclusion criteria of participants, and any risk of biases in selection of the subjects.

This SR found heterogeneity between studies regarding the length of DIT measured. Therefore, this SR has identified the need for more clarity on how long DIT should be measured in order to provide accurate results, and in order to achieve more homogenous study designs in this field.

This SR also highlights the heterogeneous ways DIT is reported between studies (kJ or kcal, % of ECM, or % above baseline RMR). It is recommended that future studies provide DIT in all of these three units of measure (kJ or Kcal, % ECM, and % above baseline RMR) in order to allow easier comparison with other studies conducted in this field and future meta-analyses.

It is also recommended for future studies to provide the data regarding baseline RMR and energy content provided by the meal, as this will allow a more comprehensive picture of the study design and results.

The majority of the studies had very small sample sizes. It is therefore recommended that future studies either increase the sample size in order to improve the statistical power of the studies, or provide evidence that the sample size used was adequate to detect an effect.

## 5. Conclusions

This systematic review has consolidated the current evidence regarding the effects of variations in energy intake, macronutrient composition, and the pattern of meals consumption after an overnight fast on DIT. It has also identified a substantial number of questions that remain to be answered, and the high level of uncertainty around many of the influences on the effects of meals on DIT. There is an enormous scope for future high quality studies in this field of research. Consensus on the duration of DIT measurement and larger sample sizes are just two ways in which research in this area could be improved. Comparisons of the effects of manipulations of meals consumed after an overnight fast on DIT in males and females, different age groups, and those who are healthy or have a range of obesity-related health conditions would also be informative.

## Figures and Tables

**Table 1:** Participant characteristics and study protocols.

Reference and Location	Sample (*n*) Males (*m*) % Age (Years)	BMI (kg/m^2^) FFM (% or kg) FM (% or kg)	RMR (kJ/h, Measured before Interventions a, b, c, etc.)	Protocol	Gap between Intervention Meals and Types of Meals Provided
**Higher vs. lower energy intake**					
Kinabo and Durnin [31]—Paper A ^††^ UK	*n* = 16 *m* = 0 ^†^ % 22 (5.8) ^†^ years	BMI = 20.8 (0.2) ^†^ FFM = 42.9 (3.1) ^†^ kg FM = 240 (23.2) ^†^ g/kg body weight	(a) 222 ^†^ (69.7) ^†^ (b) 219 ^†^ (69.7) ^†^ (c) 221.4 ^†^ (69.7) ^†^ (d) 208.2 ^†^ (69.7) ^†^	Arrival at 8:00 a.m.; fasting from 8:00 p.m. 30 min rest; RMR measured, meal consumed within 10 min.	Gap = each meal provided on different days. (a) high CHO, low Fat—lower energy (b) low CHO, high Fat—lower energy (c) high CHO, low Fat—higher energy (d) low CHO, high Fat—higher energy
Hill et al. [23] USA	*n* = 8 (4 low VO_2_max group average is 43 mL/kg/min—4 high VO_2_max group average is 62 mL/kg/min) *m* = 100 ^†^ % high VO_2_max 20 (3.5) ^†^ years low VO_2_max 26 (5.2) ^†^ years	High VO_2_max BMI = 21.9 ^†^ FFM = 62.9 (3.6) ^†^ kg FM = 13.8 (2.8) ^†^ % Low VO_2_max BMI = 23.7 ^†^ FFM = 66.8 (6.9) ^†^ kg FM = 16 (4.2) ^†^ %	High VO_2_max subjects 301.7 ^†^ (22.5) ^†^ Low VO_2_max subjects 294.6 ^†^ (40.5) ^†^	12 h fast; rest for 60–90 min; then RMR measured; meals consumed within 10 min.	Gap = NPI Liquid meal (a) lower energy intake (b) medium level energy intake (c) higher energy intake
Martin et al. [36] France	*n* = 10 *m* = 100 ^†^ % 28 (2) years	BMI = 22.2 (0.5)		14 days of intervention meals: meal consumed at research centre daily between 7:00 and 8:00 a.m. On day 15: arrive at 7:00 a.m.; overnight fast; RMR measured; meal consumed within 30 min at 8:00 a.m.	Gap = 28 days (a) 14 days of intervention meals: low energy, moderate fat breakfast (b) 14 days of intervention meals: high energy, low fat breakfast
Bennet et al. [21] USA	*n* = 4 (untrained) *m* = 100 ^†^ % 28 (8) years Excluded trained subjects	BMI = 23 ^†^ (3) ^†^ FM = 20.5 ^†^ (4.3) ^†^ %		Meal consumed at 8:30 a.m.	Gap ≥ 24 h (a) normal meal provided (25% of energy intake of the total day) (b) high fat meal: plus 50 g of fat compared to the normal fat meal
Segal et al. [22] USA	*n* = 11 *m* = 100 ^†^ % 31 (6.3) ^†^ years Excluded obese subjects	BMI = 25.5 ^†^ FFM = 66.1 (4.1) ^†^ kg FM = 15.3 (2.2) ^†^ %	343.9 ^†^ (31.6) ^†^	12 h fast; 9:00 a.m. arrival; 30 min rest; RMR measured for three five-minute measurements.	Gap = NPI (a) 35% of each man 24 h RMR meal (b) 3013 kJ meal
**Meals varying in macronutrients composition**					
Nagai et al. [30] Japan	*n* = 14 *m* = 100 ^†^ % 23.6 (1.8) ^†^ years	BMI = 21.3 (1.4) ^†^ FM = 18.4 (3.6) ^†^ %	375.3 ^†^ (39.7) ^†^ 373.2 ^†^ (44.0) ^†^	Fast from 10:00 p.m.; arrival at 7:30 a.m.; rest for 30 min; continuous RMR measurement. (NP length); meals consumed at 8:30 a.m. within 15 min.	Gap = NPI (a) standard meal—low fat meal (b) standard meal—high fat meal
Blundell, Cooling and King [33] UK	*n* = 24 *m* = 100 ^†^ % High fat consumers (*n* = 12) 20.7 (1.6) ^†^ years Low fat consumers (*n* = 12) 21.6 (2.3) ^†^ years	High fat consumers BMI = 21.2 (5.0) ^†^ FM = 11.1 (4.3) ^†^ % Low fat consumers BMI = 22.4 (2.0) ^†^ FM = 11.4 (4.3) ^†^	High fat consumers 286.08 ^†^ (0.02) ^†^ Low fat consumers 259.58 ^†^ (0.03) ^†^	Arrival at ~9:00 a.m. after 12 h fast; 30 min of steady RMR were measured; milkshake consumed within 5 min.	Gap = NPI (a) high fat milkshakes drink (b) high CHO milkshake drink
Bowden and McMurray [20] USA	*n* = 6 *m* = 0 ^†^ % 33.8 (10.7) years Excluded trained subjects	BMI = 21.7 (1.6) FFM = 43.9 (3.3) Kg FM = 21.4 (3.7) %	208.2 ^†^ (12.6) ^†^	Arrival at lab at 6:30 a.m.; 10 h fast; RMR obtained over two 5 min periods; meal consumed within 20 min	Gap = 2 days (a) high CHO meal (b) high fat meal
Thyfault et al. [25] USA	*n* = 12 Sedentary *m* = 100 ^†^ % 24.8 (4.6) ^†^ years Excluded trained subjects	FM = 21.5 (4.3) ^†^ % FFM = 58.3 (2.7) ^†^ kg	7262 (394.7) kJ ^†^—NP unit of time	Arrival at 5:00 a.m.; 12 h fast; 30 min supine rest; RMR measured for 30 min; meal consumed within 10 min.	Gap = 7 days (a) high carbohydrate liquid meal (b) moderate fat liquid meal
Raben et al. [15] Denmark	*n* = 19 *m* = 52.6 ^†^ % 23.3 (2.1) ^†^ years	BMI = 22.1 (1.7) ^†^ FM = 18.8 (4.7) ^†^ %		Arrival at 8:00 a.m.; 10 h fast; 30 min supine rest; RMR measured for 45 min; meals consumed within 15 min at 9:45 a.m.	Gap = ≥4 weeks and no more than 8 weeks standard meal (a) high protein meal (b) high fat meal (c) high CHO meal Excluded high alcohol meal
Petzke and Klaus [38] Germany	*n* = 6 *m* = 0 ^†^ % 25.5 (2.6) years	BMI = 20.6 (2.5)	(a) 218 (12) (b) 230 (13)	12 h fast; RMR measured for 30 min between 8 and 9 a.m.; meal ingested between 9 and 9:30 a.m. and within 10 min.	Gap = 2 days (a) low protein meal (b) adequate protein meal
Riggs et al. [24] USA	*n* = 21 *m* = 0 ^†^ % Overweight (*n* = 6) 22.8 (2.4) ^†^ years Normal weight (*n* = 12) 20.8 (2.6) ^†^ Underweight (*n* = 3) 20.7 (2.2) ^†^ years	Overweight BMI = 26.9 (1.7) ^†^ FFM = 48.4 (3.9) ^†^ kg FM = 31.4 (2.7) ^†^ % Normal weight BMI = 21.1 (1.7) ^†^ FFM = 44.6 (4.0) ^†^ kg FM = 23.0 (2.9) ^†^ % Underweight BMI = 18.1 (1.5) ^†^ FFM = 41.7 (4.0) ^†^ kg FM = 19.5 (2.4) ^†^ %		12 h fast; 10 min rest; RMR measured between 7 and 8:00 a.m.; meal eaten within 15–20 min.	Gap = 1 week to 2.5 months (a) high protein, high fat bars (b) high protein, low fat bars
Clegg et al. [17] UK	*n* = 7 *m* = 14.3 ^†^ % 25.7 (3.6) years	BMI = 21.9 ^†^		Arrived after an overnight fast; rested for 30 min; RMR measured between 7:30 a.m. to 9:00 a.m. at 1 min intervals for 30 min; meal consumed within 15 min.	Gap = minimum of four days (a) meal with bell pepper and sunflower oil (18.4 g) (b) meal with bell pepper and MCT oil (20.0 g) Excluded two chilli meals
Kasai et al.—study 1 [14] Japan	*n* = 8 *m* = 100 ^†^ % 26.8 (1.9) ^†^ years	BMI = 22.7 (2.1) ^†^	(a) 294.6 ^†^ (32.3) ^†^ (b) 280.3 ^†^ (29.9) ^†^ (c) 286.3 ^†^ (23.5) ^†^	Dinner at 9:00 p.m.; overnight fast; meal consumed at 11:00 a.m.	Gap = NP Liquid meal with (a) 10 g LCT (b) 5 g MCT; 5 g LCT (c) 10 g MCT
Kasai et al.—study 2 [14] Japan	*n* = 8 (*n* = 7 for the two mayonnaise arms as one drop out) *m* = 0 ^†^ % 28.1 (3.7) ^†^ years	BMI = 18.8 (1.1) ^†^	(a) 211.1 ^†^ (14.9) ^†^ (b) 206.2 ^†^ (22.5) ^†^ (c) 209.4 ^†^ (34.9) ^†^ (d) 198.2 ^†^ (32.8) ^†^	Dinner at 9:00 p.m.; overnight fast; meal consumed at 11:00 a.m.	Gap = 1 to 2 day interval within the same week for each experimental session (mayonnaise trials and margarine trials) Standard meal with (a) mayonnaise with 5 g LCT (b) mayonnaise with 5 g MCT (c) margarine with 5 g LCT (d) margarine with 5 g MCT
Casas-Agustench et al. [39] Spain	*n* = 29 *m* = 100 ^†^ % 22 (4) years	BMI = 24.1 (4.5)	(a) 318.5 (95% CI 298.2—338.8) (b) 318.9 (95% CI 298.5—339.3) (c) 323.2 (95% CI 302.3—344.1)	Arrived at 8:00 a.m. fast; 10 min rest; RMR measured for 30 min; meal provided at 9:00 a.m. and eaten within 30 min	Gap = 1–11 days (a) standard meal rich in PUFA (b) standard meal rich in MUFA (c) standard meal rich in SFA
Piers et al. [18] Australia	*n* = 14 *m* = 100 ^†^ % 38 (9) years	BMI = 27.8 (3.2) FFM = 62.7 (8.5) kg FM = 29.5 (4.8) %	(a) 311 (40) (b) 307 (36)	Arrival at 7:00–8:00 a.m.; 12–14 h of fast; 30 min rest; RMR measured for 35 min.	Gap = 7–14 days (a) meal with SFA (b) meal with MUFA
Bendixen et al. [37] Denmark	*n* = 11 *m* = 100 ^†^ % 25.1 (1.6) ^†^ years	BMI = 22.5 (1.9) ^†^ FM = 18 (3.2) ^†^ % FFM = 62.9 (6.3) ^†^ kg	(a) 290.4 ^†^ (28.5) ^†^ (b) 289.8 ^†^ (22.8) ^†^ (c) 292.2 ^†^ (26.6) ^†^ (d) 289.8 ^†^ (34.2) ^†^	Fast ≥ 12 h; 30 min supine rest; RMR measured for 45 min; meal consumed by 15 min.	Gap = 14–28 days Standard meal with liquid test drink (a) conventional fat (rapeseed oil) (b) chemically structured fat (rapeseed oil and octanoic acid by esterification with sodium methoxide) (c) lipase structured fat (rapeseed oil and octanoic acid by esterification with lipoxime IM) (d) physically mixed fat (blending rapessed oil and trioctanoate)
**Processed vs. unprocessed meals**					
Barr and Wright [26] USA	*n* = 17 in analyses *m* = 29.4 ^†^ % 25.5 (12.4) ^†^ years	BMI = 22.0 (2.2) ^†^		Fast for 12 h; 2 RMR measurements ~30 min apart before and just before consuming the meal; meals consumed between 9:15 and 11:15 a.m. and at approximately the same time for each measurement sessions.	Gap = on two consecutive days or not longer than a week apart. (a) whole-food meal as either 1½ sandwich or 2 sandwiches (b) pre-prepared processed foods as either 1½ sandwich or 2 sandwiches.
**Bolus vs. smaller frequent meals**					
Kinabo and Durnin [32]-Paper B Scotland, UK	*n* = 18 *m* = 0 ^†^ % Group A (*n* = 8) 24 (5.3) ^†^ years Group B (*n* = 10) 20 (7.2) ^†^ years	Group A BMI = 21 (1.3) ^†^ FFM = 42.4 (2.9) ^†^ kg FM = 23 (2.6) ^†^ % Group B BMI = 21 (2.1) FFM = 43.6 (4.2) ^†^ kg FM = 23 (6) ^†^ %	Group A: (a) 226.8 ^†^ (27.0) ^†^ (b) 214.8 ^†^ (20.6) ^†^ Group B (c) 230.4 ^†^ (30.6) ^†^ (d) 221.4 ^†^ (14.4) ^†^	Arrival at 8:00 a.m., at least 12 h fast; 30 min supine rest; RMR measured; meal consumed either as a large bolus meal within 20 min or as two smaller meals within 10 min every 180 min.	Gap = 1 week Group A: (a) high carb-low fat meal; one large meal (b) high carb-low fat meal; two smaller meals Group B: (c) low carb-high fat meal; one large meal (d) low carb-high fat meal; two smaller meals
Vaz et al. [34] Australia	*n* = 10 *m* = 100 ^†^ %	BMI = 22.9 (1.8) ^†^ FFM = 64 (5.7) ^†^ kg FM = 16.6 (6.0) ^†^ %		12–14 h overnight fast; RMR measured after 30 min rest.	Gap = approximately 14 days (a) standard meal-single meal (b) standard meal- three smaller meals
Allirot et al. [35] France	*n* = 20 *m* = 100 ^†^ % 27.1 (5.7) ^†^ years	BMI = 22.0 (1.3) ^†^		Arrival at 7:00 a.m.; fast since 9:00 p.m.; RMR measured for 30 min; meals consumed either as a bolus event within 20 min or as smaller meals every hour within 10 min each.	Gap = at least 7 days (a) one 20 min long episode (b) 4 smaller meals in 10 min episodes
Tai et al. [27] USA	*n* = 7 *m* = 0 ^†^ % 26.7 (2.9) years	BMI = 20.8 (2.1) FM = 17.1 (5.4) %	(a) 233.7 ^†^ (9.8) ^†^ (b) 236.22 ^†^ (14.1) ^†^	RMR measured after 12–14 h fast and minimum of 30 min rest; meals consumed as one bolus event within 10 min or as smaller meals every 30 min.	Gap = meals provided on different days (a) liquid meal taken in one eating event of 10 min long (b) liquid meal in 6 equal smaller meals at 30 min interval over 150 min.
**Fast vs. slow eating patterns**					
Hamada et al. [28] Japan	*n* = 10 *m* = 100 ^†^ % 25 (1) years	BMI = 19.8 ^†^ FM = 13 (2) %		Fast since dinner (>10 h); 20 min semi-supine position rest; RMR measured for 20 min.	Gap = NPI standard meal (a) rapid eating (b) slow eating
Toyama et al. [29] Japan	*n* = 9 *m* = 0% 22 (2.1) years	BMI (a) 21.3 (1.7) (b) 21.3 (1.8) FM (a) 24.1 (3.8) % (b) 24.0 (4.0) %	(a) 196.8 ^†^ (17.3) ^†^ (b) 191.6 ^†^ (17.6) ^†^	Dinner by 9:00 p.m., fast until morning; arrival at 8:00 a.m., 30 min supine rest; RMR measured; meal consumed at 9:00 a.m.	Gap = at least 7 days Same meal provided (a) fast eating (5 min) (b) regular eating (15 min)
**Palatable vs. unpalatable meals**					
Sawaya et al. [19] USA	*n* = 19 *m* = 100 ^†^ % Old (*n* = 9) 69.4 (1.3) years Young (*n* = 10) 23.4 (1) years	Old BMI = 24.4 (0.9) FFM = 55 (2.2) FM = 26.2 (1.9) % Young BMI = 22.7 (0.5) FFM = 64.1 (1.9) kg FM = 12 (1.3) %	(a) young 319.8 ^†^ (32.4) ^†^ old 280.2 ^†^ (30.5) ^†^ (b) young 319.2 ^†^ (32.4) ^†^ old 268.8 ^†^ (23.8) ^†^	Sleep at university by 10:00 p.m., awakened at 6:30 a.m.; 30 min rest; RMR measured for 30 min; meals consumed within 20 min.	Gap = 1 week interval (a) palatable meal (b) control meal
Weststrate et al. [16] Netherlands—study 1	*n* = 12 *m* = 50 ^†^ % Men 22.7 (1.8) ^†^ years Women 21.2 (1.8) ^†^ years	Men FM = 12.0 (2.2) ^†^ % Women FM = 29.0 (2.5) ^†^ %		Overnight fast.	Gap = At least 2 days (a) palatable meal (b) unpalatable meal

Data are described in mean (SD) unless otherwise indicated. ^†^ These data (mean and/or SD) were calculated or converted for one or more of these possible calculations or conversions (calculated the average and/or SD from individuals’ data; kcal converted to kJ; MJ converted to kJ; RMR kJ converted for unit of time; SE converted to SD, males’ percentage calculated from the total number of males in the sample). ^††^ Kinabo et al. [31]—paper A is also part of results section: “meals varying in macronutrient composition”. a, b, c or d: refers to the different types of interventions provided as explained in the last column (gap between intervention meals and types of meals provided). BMI = Body Mass Index. CHO = Carbohydrate. FM = Fat Mass. FFM = Fat Free Mass. LCT = Long Chain Triglycerides. M = Males. MCT = Medium Chain Triglycerides. MUFA = Mono Unsaturated Fatty Acids. N = Sample. NP = Not Provided. NPI = Not Provided Information. PUFA = Poly Unsaturated Fatty Acids. RMR = Resting Metabolic Rate. SFA = Saturated Fatty Acids. VO_2_ = Rate of Oxygen consumption.

**Table 2:** Consumption of meals after an overnight fast and DIT.

Reference and DIT Measurement	Energy Ingested (kJ) and % Energy from Macronutrients	DIT (kJ)	DIT % Energy Content of the Meal	DIT % above Baseline	Conclusions
**Higher vs. lower energy intake**					
**Kinabo and Durnin** [31] **Paper A** ^††^ DIT = open circuit indirect calorimetry using Douglas bag for 5 h; DIT measured for 10 min, every 10 min for 3 collections then every 20 min for 5 collections	(a and b) 2520 kJ (c and d) 5040 kJ (a and c) 70% CHO, 11% protein, 19% fat (b and d) 24% CHO, 11% protein and 65% fat	(a) 45.6 ^†^ (9.3) ^†^ kJ/h (b) 45.6 ^†^ (10.8) ^†^ kJ/h (c) 71.2 ^†^ (15.5) ^†^ kJ/h (d) 68 ^†^ (12.4) ^†^ kJ/h NS (a and c vs. b and d for kJ/5 h) *** (a and b vs. c and d for kJ or kcal /5 h) NS (all four meals compared for kJ or kcal /5 h)	(a) 1.8 ^†^ (0.4) ^†^ %/h (b) 1.8 ^†^ (0.5) ^†^ %/h (c) 1.4 ^†^ (0.3) ^†^ %/h (d) 1.4 ^†^ (0.2) ^†^ %/h	(a) 21 (4.3) ^†^% (b) 21 (5.8) ^†^ % (c) 33 (7.7) ^†^ % (d) 33 (6.2) ^†^ %	No significant difference between meals differing on macronutrient compositions. Significantly higher DIT for meals with higher energy intake compared to lower energy intake.
**Hill et al.** [23] DIT = indirect calorimetry for 3 h; DIT measured every 30 min	(a) 2092 kJ ^†^ (b) 4184 kJ ^†^ (c) 6276 kJ ^†^ 50% CHO, 16% protein, 34% fat			(a) high and low VO_2_ group: less than 10% (b) high VO_2_max 23% low VO_2_max 19% (c) high VO_2_max 41%, low VO_2_max 26% *p* value NP	An increase in meal size increased DIT. Unclear if effect was significant.
**Martin et al.** [36] DIT = ventilated hood indirect calorimetry for 4 h; DIT measured every hour	(a) 418 kJ, 62% CHO, 34.4% fat, 3.6% protein (b) 2920 kJ, 67% CHO, 24.6% fat, 8.4% protein	(a) 4.5 ^†^ (1.4) ^†^ kJ/h (b) 35.6 ^†^ (2.6) ^†^ kJ/h	(a) 1.1 (0.3) %/h (b) 1.2 (0.1) %/h *p* value NP		No difference on DIT between low energy, moderate fat meal, and high energy, low fat meal.
**Bennet et al.** [21] DIT = ventilated hood indirect calorimetry for 6 h; continuously	NP kJ (a) 55% CHO, 30% fat, 15% protein (b) same meal as above plus 50 g of fat (addition of 1881 kJ compared to breakfast (a))		(a) 1.3 ^†^ (0.3) ^†^ %/h (b) 1.2 ^†^ (0.40) ^†^ %/h NS (a vs. b for all subjects (trained and untrained) only included untrained for this SR)		No significant difference in DIT between high fat meal and normal fat meal for the overall subjects (trained vs. untrained).
**Segal et al.** [22] DIT = open circuit indirect calorimetry for 3 h; DIT measured for at least 6 min periods every 30 min	(a) 35% of each man 24 h RMR (2889 *^‡^ kJ) NP macronutrients (b) 3013 kJ ^†^, 55% CHO, 24% protein, 21% fat	(a) 89.3 ^†^ (17.6) ^†^ kJ/h (b) 96.3 ^†^ (17.6) ^†^ kJ/h	(a) 3.2 ^†^ (0.8) ^†^ %/h (b) 3.3 ^†^ (0.4) ^†^ %/h NS (a vs. b %/3 h)	(a) 11.9% (b) 12.9%	N.A.
**Meals varying in macronutrient composition**					
**Nagai et al.** [30] DIT = open circuit indirect calorimetry for 3.5 h; DIT measured for 6 min every 30 min	3255 (306.5) ^†^ kJ (a) 70% CHO, 10% protein, 20% fat (b) 20% CHO, 10% protein, 70% fat	(a) 43.1 ^†^ (13.7) ^†^ kJ/h (b) 32.6 ^†^ (14.1) ^†^ kJ/h * (a vs. b for kJ/3.5 h)	(a) 1.3 ^†^ (0.4) ^†^ %/h (b) 1.0 ^†^ (0.4) ^†^ %/h * (a vs. b for %/3.5 h ECM)	(a) 1.7 (0.7) ^†^ % (b) 1.3 (0.4) ^†^ % * (a vs. b for % AB)	DIT was significantly higher in low fat meal compared to high fat meal.
**Blundell et al.** [33] DIT = ventilated hood indirect calorimetry for 3 h continuously	2092 kJ (a) 19.9% CHO, 78.8% fat, 1.3% protein (b) 90.4% CHO, 1.3% fat, 8.3% protein	(a) high fat consumers 27.5 ^†^ (28.9) ^†^ kJ/h; low fat consumers 25.6 ^†^ (14.5) ^†^ kJ/h (b) high fat consumers 38.2 ^†^ (26.0) ^†^ kJ/h; low fat consumers 35.2 ^†^ (15.6) ^†^ KJ/h * (a vs. b for kJ/day)	(a) high fat consumers 1.3 ^†^ (1.4) ^†^ %/h; low fat consumers 1.2 ^†^ (0.7) ^†^ %/h (b) high fat consumers 1.7 ^†^ (1.2) ^†^ %/h; low fat consumers 1.7 ^†^ (0.7) ^†^ %/h	(a) high fat consumers 10.2 ^†^ (10.8) ^†^ % low fat consumers 9.9 ^†^ (5.6) ^†^ % (b) high fat consumers 14.2 ^†^ (9.7) ^†^ % low fat consumers13.6 ^†^ (6.0) ^†^ %	The consumption of a high-carbohydrate meal was significantly associated with an increased DIT compared to a high fat meal
**Bowden and McMurray** [20] DIT = open circuit spirometry for 5 h; DIT measured for 10 min periods every 30 min	(a) 2068 kJ, 76% CHO, 5% protein, 23% fat (b) 2093 kJ (fixed amount), 21% CHO, 8% protein, 72% fat	(a) 54.6 ^†^ kJ/h (b) 27.8 ^†^ kJ/h	(a) 2.6 ^†^ %/h (b) 1.3 ^†^ %/h *p* value NP	(a) 26.2 ^†^ % (b) 13.4 ^†^ %	No significant difference on total energy expenditure between high CHO and high fat meal.
**Thyfault et al.** [25] DIT = indirect calorimetry with face mask for 4 h; DIT measured continuously with measurements averaged over 15 min periods for 1 h then for 30 min periods for the remaining hours	(a) 3021 (1194.0) ^†^ kJ, 79% CHO, 20% protein, 1% fat (b) 2996 (1167.4) ^†^ kJ, 37% CHO, 18% protein, 45% fat	(a) 57.8 ^†^ (19.1) ^†^ kJ/h or 1.0 (0.3) ^†^ kJ/FFM/h or 0.7 (0.9) ^†^ kJ/BM/h (b) 49.8 ^†^ (21.6) ^†^ kJ/h or 0.8 (0.3) ^†^ kJ/FFM/h or 0.6 (0.9) ^†^ kJ/BM/h *p* value NP	(a) 1.9 ^†^ (0.6) ^†^ %/h (b) 1.7 ^†^ (0.7) ^†^ %/h *p* value NP		N.A.
**Raben et al.** [15] DIT = indirect calorimetry with an open-circuit ventilated hood system; continuously for 5 h with 5 min breaks every h if needed	2500 kJ for f, 3000 kJ for m (a) 37.2% CHO, 31.8% protein, 31.1% fat (b) 23.9% CHO, 11.6% protein, 64.6% fat (c) 65.4% CHO, 12.2% protein, 23.7% fat	(a) 45.9 ^†^ kJ/h (b) 39.2 ^†^ kJ/h (c) 39.2 ^†^ kJ/h	(a) 1.7%/h (b) 1.4%/h (c) 1.4%/h ** (a vs. b vs. c vs. also meal with alcohol excluded for this SR for %/5 h)		Significant difference in DIT between the different meal types administered. Protein had a higher DIT compared to fat and CHO meals.
**Petzke and Klaus** [38] DIT = indirect calorimetry ventilated-hood system for 6 h; 3 × 30 min measurements (first 5–10 min discarded) at 30, 150, and 270 min	(a) 3114 kJ, 35.4% CHO, 3.9% protein, 60.7% fat (b) 3131 kJ, 27.8% CHO, 11.4% protein, 60.8% fat	(a) 7.8 ^†^ (1.0) ^†^ kJ/h (b) 22.4 ^†^ (5.7) ^†^ kJ/h *** (a vs. b for kJ/6 h)	(a) 1.5 ^†^ (0.2) ^†^ %/h (b) 4.3 ^†^ (1.1) ^†^ %/h *** (a vs. b for %/6 h)	(a) 3.6 ^†^ (0.5) ^†^ % (b) 9.7 ^†^ (2.5) ^†^ %	DIT was significantly higher in adequate protein meal compared to low protein meal.
**Riggs et al.** [24] DIT = indirect calorimetry for 3.5 h; DIT measured every 30 min	1841 kJ ^†^ (a) 23% CHO ^†††^; 34% protein, 43% fat (b) 48% CHO ^†††^; 28% protein, 24% fat	Overweight (a) 48.4 ^†^ (20.0) ^†^ kJ/h (b) 46.3 ^†^ (18.8) ^†^ kJ/h Normal weight (a) 43.1 ^†^ (19.2) ^†^ kJ/h (b) 31.0 ^†^ (19.2) ^†^ kJ/h Underweight (a) 20.0 ^†^ (16.4) ^†^ kJ/h (b) 24.2 ^†^ (15.6) ^†^ kJ/h ** (a vs. b for normal subjects for kcal/min/kg FFM)	Overweight (a) 2.6 ^†^ (0.2) ^†^ %/h (b) 2.5 ^†^ (1.0) ^†^ %/h Normal weight (a) 2.3 ^†^ (1.0) ^†^ %/h (b) 1.7 ^†^ (1.0) ^†^ %/h Underweight (a) 1.1 ^†^ (0.9) ^†^ %/h (b) 1.3 ^†^ (0.9) ^†^ %/h ** (a vs. b for normal subjects for %/3.5 h)		Significantly higher DIT for the high protein, high fat meal in normal weight subjects.
**Clegg et al.** [17] DIT = ventilated hood indirect calorimetry for 6 h; measured for 15 min every 30 min	1863 kJ 35.5% CHO, protein 19.9%, 44.6% fat	(a) 21.9 ^†^ (7.9) ^†^ kJ/h (b) 29.4 ^†^ (8.4) ^†^ kJ/h ** (a vs. b for kcal/6 h)	(a) 1.2 ^†^ (0.4) ^†^ %/h (b) 1.6 ^†^ (0.5) ^†^ %/h ** (a vs. b for %/6 h)		Pepper sunflower oil had a significantly lower DIT than pepper MCT oil intervention.
**Kasai et al.** [14] **study 1** DIT = indirect calorimetry Aeromonitor AE-300S; DIT measured for 6 h at 1 h intervals	(a) 1046 kJ ^†^ (b) 1029 kJ ^†^ (c) 1013 kJ ^†^ 43% CHO, 21% protein, 36% fat	(a) 8.4 ^†^ (4.6) ^†^ kJ/h or 0.1 ^†^ (0.07) ^†^ kJ/kg/h (b) 17.7 ^†^ (10.8) ^†^ kJ/h or 0.3 ^†^ (0.2) ^†^ kJ/kg/h (c) 19.5 ^†^ (14.1) ^†^ kJ/h or 0.3 ^†^ (0.2) ^†^ kJ/kg/h ** (a vs. b for cal/kg/6 h) ** (a vs. c for cal/kg/6 h) NS (b vs. c for cal/kg/6 h)	(a) 0.8 ^†^ (0.5) ^†^ %/h (b) 1.7 ^†^ (1.1) ^†^ %/h (c) 1.9 ^†^ (1.4) ^†^ %/h ** (a vs. b for %/5 h) ** (a vs. c for %/5 h) NS (b vs. c for kJ/5 h)	(a) 2.8 ^†^ (1.7) ^†^ % (b) 6.3 ^†^ (3.9) ^†^ % (c) 7.3 ^†^ (5.2) ^†^ %	Significant increase in DIT when a liquid meal containing MCT was consumed compared to a meal with LCT.
**Kasai et al.** [14] **study 2** DIT = indirect calorimetry Aeromonitor AE-300S; DIT measured for 6 h at 1 h intervals	(a) 1059 kJ ^†^ (b) 1042 kJ ^†^ (c) 1020 kJ ^†^ (d) 1004 kJ ^†^50% CHO, 10% protein, 40% fat	(a) 8.2 ^†^ (6.4) ^†^ kJ/h or 0.2 ^†^ (0.1) ^†^ kJ/kg/h (b) 14.0 ^†^ (5.7) ^†^ kJ/h or 0.3 ^†^ (0.1) ^†^ kJ/kg/h (c) 9.8 ^†^ (8.2) ^†^ kJ/h or 0.2 ^†^ (0.2) ^†^ kJ/kg/h d) 20.3 ^†^ (15.7) ^†^ kJ/h or 0.4 ^†^ (0.3) ^†^ kJ/kg/h * (a vs. b for cal/kg/6 h) * (c vs. d for cal/kg/6 h)	(a) 0.8 ^†^ (0.6) ^†^ %h (b) 1.3 ^†^ (0.5) ^†^ %h (c) 1.0 ^†^ (0.8) ^†^ %h (d) 2.0 ^†^ (1.6) ^†^ %h * (a vs. b for %/5 h) * (c vs. d for %/5 h)	(a) 3.9 ^†^ (3.0) ^†^ % (b) 6.8 ^†^(2.7) ^†^ % (c) 4.7 ^†^ (3.9) ^†^ % (d) 10.5 ^†^ (7.9) ^†^ %	Significant increase in DIT in meals containing mayonnaise or margarine with MCT compared to LCT.
**Casas-Agustench et al.** [39] DIT = open circuit indirect calorimetry with a canopy system for 5 h continuously	Mean (95% CI) (a) 2655 (2510–2799) kJ, 36.4 (35.9–36.7)% CHO, 11.7 (11.4–11.9)% protein, 51.9 (95% CI 51.7–52.1)% fat (b) 2608 (2428–2788) kJ, 37 (95% CI 36.6–37.4)% CHO, 11.3 (95% CI 10.6–11.9)% protein, 51.7 (95% CI 51.3–52.0)% fat (c) 2599 (2421–2278) kJ, 37.1 (95% CI 36.4–37.7)% CHO, 11.2 (95% CI 10.8–11.6)% protein, 51.7 (95% CI 51.2–52.1)% fat	Mean (95% CI) (a) 37.2 ^†^ (29.5–44.8) ^†^ kJ/h (b) 36.8 ^†^ (30.5–43.0) ^†^ kJ/h (c) 30.0 ^†^ (24.2–35.8) ^†^ kJ/h * (a vs. b vs. c and a vs. c for kJ/5 h)	Mean (95% CI): (a) 1.4 ^†^ (1.1–1.7) ^†^ %/h (b) 1.4 ^†^ (1.2–1.7) ^†^ %/h (c) 1.2 ^†^ (0.9–1.4) ^†^ %/h	Mean (95% CI) (a) 12.3 (9.7–14.9)% (b) 11.8(9.7–13.9)% (c) 9.6 (7.7–11.4)% * (a vs. c and b vs. c for %AB) * (a vs. b vs. c for % AB)	DIT was significantly higher in PUFA and MUFA meals compared to SFA meal.
**Piers et al.** [18] DIT = open circuit ventilated hood canopy system for 5 h; DIT measured for 30 min periods each hour	2500 ^†^ kJ 42% CHO, 15% of energy from protein, 43% fat	(a) 29.6 ^†^ (10) ^†^ kJ/h (b) 28.4 ^†^ (10) ^†^ kJ/h NS (a vs. b for kJ/5 h)	(a) 1.2 ^†^ (0.4) ^†^ %/h (b) 1.1 ^†^ (0.4) ^†^ %/h NS (a vs. b for %/5 h)	(a) 9.5 ^†^ (3.2) ^†^ % (b) 9.3 ^†^ (3.2) ^†^ %	No significant differences in DIT between SFA and MUFA meals.
**Bendixen et al.** [37] DIT = indirect calorimetry with open circuit, ventilated hood for 5 h continuously with 10 min breaks every hour	4698 (550.2) ^†^ kJ, 34% CHO, 6% protein, 60% fat	(a) 61.8 ^†^ (15.2) ^†^ kJ/h (b) 72.8 ^†^ (19.0) ^†^ kJ/h (c) 69.2 ^†^ (11.4) ^†^ kJ/h (d) 65 ^†^ (13.9) ^†^ kJ/h * (a vs. b vs. c vs. d for kJ/5 h) ** (a vs. b for kJ/5 h) NS (All other pairwise comparison apart from a vs. b)	(a) 1.3 ^†^ (0.3) ^†^ %/h (b) 1.5 ^†^ (0.3) ^†^ %/h (c) 1.4 ^†^ (0.2) ^†^ %/h (d) 1.4 ^†^ (0.3) ^†^ %/h * (a vs. b vs. c vs. d for %/5 h) ** (a vs. b for %/5 h) NS (all other pairwise comparison apart from a vs. b)	(a) 21.3 ^†^ (5.2) ^†^ % (b) 25.1 ^†^ (6.6) ^†^ % (c) 23.7 ^†^ (3.9) ^†^ % (d) 22.4 ^†^ (4.8) ^†^ %	DIT was significantly higher in the three modified fat meals compared to the conventional fat meal.
**Processed vs. unprocessed meals**					
**Barr and Wright** [26] DIT = indirect calorimetry using spirometer and gas bags (a) 5.8 (0.11) h (b) 4.8 (0.23) h 2 min measurement by spirometer followed by 10 s exhalation for 5 or 6 breaths into a gas bag every hour	2520 or 3360 kJ (a) 39% fat, 40% CHO, 20% protein (b) ½ sandwich: 33% fat, 49% CHO, 15% protein or 2 sandwiches: 33% fat, 50% CHO, 15% protein	(a) 99.4 ^†^ (40.7) ^†^ kJ/h (b) 63.9 ^†^ (35.6) ^†^ kJ/h *** (a vs. b kJ/5.8 and 4.8 h)	(a) 3.4 ^†^ (1.7) ^†^ %/h (b) 2.2 ^†^ (1.4) ^†^ %/h ** (a vs. b %/5.8 and %/4.8 h)		Whole food meal showed a significant higher DIT compared to processed meal.
**Bolus vs. smaller frequent meals**					
**Kinabo and Durnin** [32]**-Paper B** DIT = open circuit indirect calorimetry using Douglas bag technique for 6 h; DIT measured for 10 min, every 10 min for the first 90 min and every 20 min for the last 90 min.	(a and c) 5040 kJ (b and d) 2520 × 2 kJ (a and b) 70% CHO, 11% protein, 19% fat (c and d) 24% CHO, 11% protein, 65% fat	Group A: (a) 62.8 ^†^ (13.2) ^†^ kJ/h (b) 63.5 ^†^ (11.7) ^†^ kJ/h Group B: (c) 59.3 ^†^ (11.5) ^†^ kJ/h (d) 56.7 ^†^ (8.0) ^†^ kJ/h NS (a vs. b and c vs. d for kJ/6 h)	Group A (a) 1.3 ^†^ (0.3) ^†^ %/h (b) 1.3 ^†^ (0.2) ^†^ %/h Group B (c) 1.2 ^†^ (0.25) ^†^ %/h (d) 1.1 ^†^ (0.2) ^†^ %/h	Group A (a) 28 (6.9) ^†^ % (b) 31 (5.0) ^†^ % Group B (c) 27 (7.8) ^†^ % (d) 28 (5.1) ^†^ %	No significant difference on DIT between meals consumed as bolus vs. two smaller frequent meals.
**Vaz et al.** [34] DIT = indirect calorimetry for 2 h; DIT measured every 30 min	(a) 3150 ^†^ (b) 1050 × 3 kJ ^†^ 53.3% CHO, 14.7% protein, 32% fat	(a) 71 ^†^ (31.5) ^†^ kJ/h (b) 52.3 ^†^ (15.3) ^†^ kJ/h NS (a vs. b for kJ/2 h)	(a) 2.3 ^†^ (1.0) ^†^ %/h (b) 1.7 ^†^ (0.49) ^†^ %/h	(a) 22.2%/h (b) NP	DIT was lower in the small frequent feeding regime compared to one bolus meal event – but not significantly different.
**Allirot et al.** [35] DIT = indirect calorimetry for 4 h; DIT measured for 30 min periods	(a) 2823.4 kJ ^†^ (b) total 2823.4 ^†^ divided in 705.8 ^†^ kJ meals 54.2 ^†^ % CHO, 6.3 ^†^ % Protein, 36.7 ^†^ % Fat	(a) 43.8 ^†^ (18.4) ^†^ kJ/h (b) 33.2 ^†^ (15.5) ^†^ kJ/h	(a) 1.6 ^†^ (0.7) ^†^ %/h (b) 1.2 ^†^ (0.6) ^†^ %/h * (a vs. b for %/4 h)		DIT was significantly higher when the meal consumed as one bolus event compared to four smaller isocaloric meals ingested over time in the morning.
**Tai et al.** [27] DIT = indirect calorimetry for 5 h; DIT measured every 30 min	(a) one meal of 3138 kJ (b) 6 meals of 523 kJ 54.5% CHO, 14.0% protein, 31.5% fat	(a) 48.2 ^†^ (16.93) ^†^ kJ/h (b) 34.9 ^†^ (12.3) ^†^ kJ/h * (a vs. b for kJ/5 h)	(a) 1.5 ^†^ (0.5) ^†^ %/h (b) 1.1 ^†^ (0.4) ^†^ %/h	(a) 20.6 ^†^ (7.2) ^†^ % (b) 14.8 ^†^ (5.2) ^†^%	DIT was significantly higher when the meal was consumed as a one bolus event compared to six smaller isocaloric meals ingested over time in the morning.
**Fast vs. slow eating patterns**					
**Hamada et al.** [28] DIT = gas analyzer AE-310S for 1.5 h continuously	1255.2 ^†^ kJ, 42% CHO, 8% protein, 50% fat	(a) 19.5 ^†^ (142.2) ^†^ kJ/kg/h (b) 502.1 ^†^ (234.4) ^†^ kJ/kg/h * (a vs. b for kcal/kg/90 min)			Slowing eating was associated with a significant increase in DIT compared to rapid eating.
**Toyama et al.** [29] DIT = open-circuit indirect calorimetry for 3 h; first hour continuously, second and third hours measured for 15 min every 30 min intervals	1464 kJ, 61.3% CHO, 16.4% protein, 22.3% fat	(a) 31.6 ^†^ (15) ^†^ kJ/kg/h (b) 41.9 ^†^ (14.6) ^†^ kJ/kg/h NS (a vs. b for kcal/kg/min)		(a) 6.8 (4.8)% (b) 8.5 (4.2)%	There was no significant difference in DIT between fast eating and regular eating.
**Palatable vs. unpalatable meals**					
**Sawaya et al.** [19] DIT = ventilated hood indirect calorimetry, for 6 h; DIT measured for 10 min with 5 min breaks for the 6 h	2930 kJ ^†^, 65% CHO, 12% protein, 23% fat	(a) old 37.0 ^†^ (15.9) ^†^ kJ/h young 34.7 ^†^ (13.6) ^†^ kJ/h (b) old 46.4 ^†^ (18.4) ^†^ kJ/h young 39.5 ^†^ (17.0) ^†^ kJ/h	(a) old 1.3 ^†^ (0.5) ^†^ %/h young 1.2 ^†^ (0.5) ^†^ %/h (b) old 1.6 ^†^ (0.6) ^†^ %/h young 1.4 ^†^ (0.6) ^†^ %/h NS (a vs. b %/3 h)	(a) old 26.4 ^†^ (11.3) ^†^ % young 21.7 ^†^ (8.5) ^†^ % (b) old 34.6 ^†^ (13.7) ^†^ % young 24.8 ^†^ (10.6) ^†^ %	DIT did not significantly differ between palatable and unpalatable meals.
**Weststrate et al.** [16] **study 1** DIT = ventilated hood indirect calorimetry for 3.5 h continuously	2000 kJ ^†^, NP % energy from macronutrient	(a) 47.3 ^†^ (14.2) ^†^ kJ/h (b) 52.9 ^†^ (13.3) ^†^ kJ/h NS (a vs. b for kJ/3.5 h)	(a) 2.4 ^†^ (0.7) ^†^ %/h (b) 2.6 ^†^ (0.7) ^†^ %/h NS (a vs. b for %/3.5 h)		There was not a significant difference in DIT between palatable and unpalatable meals.

Data are described in mean (SD) unless otherwise described. a, b, c, etc. = these letters describe the types of meal interventions provided as illustrated in Table 1. ^†^ These data (mean and/or SD and/or 95% CI) were calculated or converted for one or more of these possible calculations or conversions (DIT % ECM calculated from DIT kJ, DIT % above baseline RMR calculated from DIT kJ, DIT kJ calculated from DIT % ECM, macronutrient % ECM calculated from grams, kcal converted to kJ, MJ converted to kJ, SE converted to SD, DIT % ECM or KJ or % above baseline RMR converted for unit of time, formulas described either in methodology or Supplementary Materials Table S1). ^††^ Kinabo et al. [31]—paper A is also part of results section: “meals varying in macronutrient composition”. ^†††^ calculated % CHO = 100 − (% energy from FAT + % of energy from protein). *^‡^ calculated 35% 24 h RMR = 35 × 8253.6 kJ (24 h RMR)/100. It is an average value. * *p* ≤ 0.05. ** *p* ≤ 0.01. *** *p* ≤ 0.001. BM = Body Mass. DIT = Diet Induced Thermogenesis. CHO = Carbohydrate. FFM = Fat Free Mass. LCT = Long Chain Triacylglycerol. MCT = Medium Chain Triacylglycerol. MUFA = Mono Unsaturated Fatty Acids. NS = Not Significant. NP = Not Provided. PUFA = Poly Unsaturated Fatty Acids. SFA = Saturated Fatty Acids. VO_2_ = Rate of Oxygen consumption.

## Supplementary Materials

The following are available online at http://www.mdpi.com/2072-6643/8/11/670/s1, Table S1: Formulas used to calculate participants’ characteristics, macronutrients compositions, or DIT.

## Abbreviations

The following abbreviations are used in this manuscript:

AB	Above Baseline
ATP	Adenosine Triphosphate
BM	Body Mass
BMI	Body Mass Index
CHO	Carbohydrate
CI	Confidence Interval
ECM	Energy Content of the Meal
FFM	Fat Free Mass
FM	Fat Mass
DIT	Diet Induced Thermogenesis
LCT	Long Chain Triglycerides
M	Male
MCT	Medium Chain Triglycerides
MUFA	Mono Unsaturated Fatty Acids
N	Sample
NPI	Not Provided Information
NP	Not Provided
NS	Not Significant
PUFA	Poly Unsaturated Fatty Acids
RCT	Randomized Controlled Trials
REE	Resting Energy Expenditure
RMR	Resting Metabolic Rate
SD	Standard Deviation
SE	Standard Error
SFA	Saturated Fatty Acids
SR	Systematic Review
VO_2_	Rate of Oxygen Consumption
