# Supplementary material for: The Energy Content and Composition of Meals Consumed after an Overnight Fast and Their Effects on Diet Induced Thermogenesis: A Systematic Review, Meta-Analyses and Meta-Regressions
Source: Nutrients. 2016 Oct 25;8(11):670. doi: 10.3390/nu8110670 (PMC5133058; doi:10.3390/nu8110670)
Supplement: Supplementary file 1 [file nutrients-08-00670-s001.docx]

Supplementary Materials: The Energy Content and Composition of Meals Consumed after an Overnight Fast and Their Effects on Diet Induced Thermogenesis: A Systematic Review, Meta-Analyses and Meta-Regressions

Angelica Quatela, Robin Callister, Amanda Patterson and Lesley MacDonald-Wicks

**Table S1.** Formulas used to calculate participants’ characteristics, macronutrients compositions, or DIT.

|  | **Formulas** |
| --- | --- |
| BMI | BMI = body weight (kg)/height (*m*)^2^ [1] |
| SD | SD = [SE × square root (sample size (*n*) − 1)] ^†^ |
| Macronutrients (KJ) | macronutrient (kJ) = macronutrient (g) × Atwater factor ^††^ |
| Percentage of energy from macronutrients | macronutrient (% of energy) = [(macronutrient (kJ)/kJ intake ^†††^) × 100] |
| DIT (kJ) | DIT (kJ) = [(DIT % ECM × kJ intake ^†††^)/100] |
| DIT % ECM | DIT % ECM = [(DIT KJ/kJ intake ^†††^) × 100] |
| DIT % AB | DIT % AB = [(DIT kJ/Fasting RMR) × 100] |

^†^ Sample size minus one was used instead of only sample size in order to account for the small sample sizes. ^††^ Atwater factors: 16 kJ for CHO, 17 kJ for protein and 37 kJ for fat. ^†††^ kJ intake for breakfast. AB = Above Baseline. DIT = Diet Induced Thermogenesis. ECM = Energy Content of Meal. RMR = Resting Metabolic Rate.

**References**

1. WHO. BMI Classification. Available online: http://apps.who.int/bmi/index.jsp?introPage=intro_3.html (accessed on 24 October 2016).
